# Exceptional topological insulators

**DOI:** 10.1038/s41467-021-25947-z

**Published:** 2021-09-28

**Authors:** M. Michael Denner, Anastasiia Skurativska, Frank Schindler, Mark H. Fischer, Ronny Thomale, Tomáš Bzdušek, Titus Neupert

**Affiliations:** 1 Department of Physics, University of Zurich, Winterthurerstrasse 190, 8057 Zurich, Switzerland; 2 Princeton Center for Theoretical Science, Princeton University, Princeton, NJ 08544 USA; 3 Institut für Theoretische Physik und Astrophysik, Universität Würzburg, 97074 Würzburg, Germany; 4 Condensed Matter Theory Group, Paul Scherrer Institute, 5232 Villigen PSI, Switzerland

**Keywords:** Electronic properties and materials, Topological insulators, Theoretical physics

## Abstract

We introduce the exceptional topological insulator (ETI), a non-Hermitian topological state of matter that features exotic non-Hermitian surface states which can only exist within the three-dimensional topological bulk embedding. We show how this phase can evolve from a Weyl semimetal or Hermitian three-dimensional topological insulator close to criticality when quasiparticles acquire a finite lifetime. The ETI does not require any symmetry to be stabilized. It is characterized by a bulk energy point gap, and exhibits robust surface states that cover the bulk gap as a single sheet of complex eigenvalues or with a single exceptional point. The ETI can be induced universally in gapless solid-state systems, thereby setting a paradigm for non-Hermitian topological matter.

## Abstract (web summary)

Three-dimensional topological insulators have become a research focal point on topological quantum matter. Here, the authors propose the non-Hermitian analogue, the exceptional topological insulator, with anomalous surface states only existing within the topological bulk embedding.

## Introduction

Since their theoretical conception^1,2^ and experimental discovery^3,4^, three-dimensional topological insulators (3D TIs) have become the focal point for research on topological quantum matter. Their key feature are conducting surface states resembling a single species of gapless Dirac electrons, which are protected against surface perturbations as long as time-reversal and charge-conservation symmetry are preserved^5^. Transcending the realm of quantum matter, the TI phase has since been realized in many different settings including meta-materials, such as photonic and phononic crystals^6–9^.

**Fig. 1:**
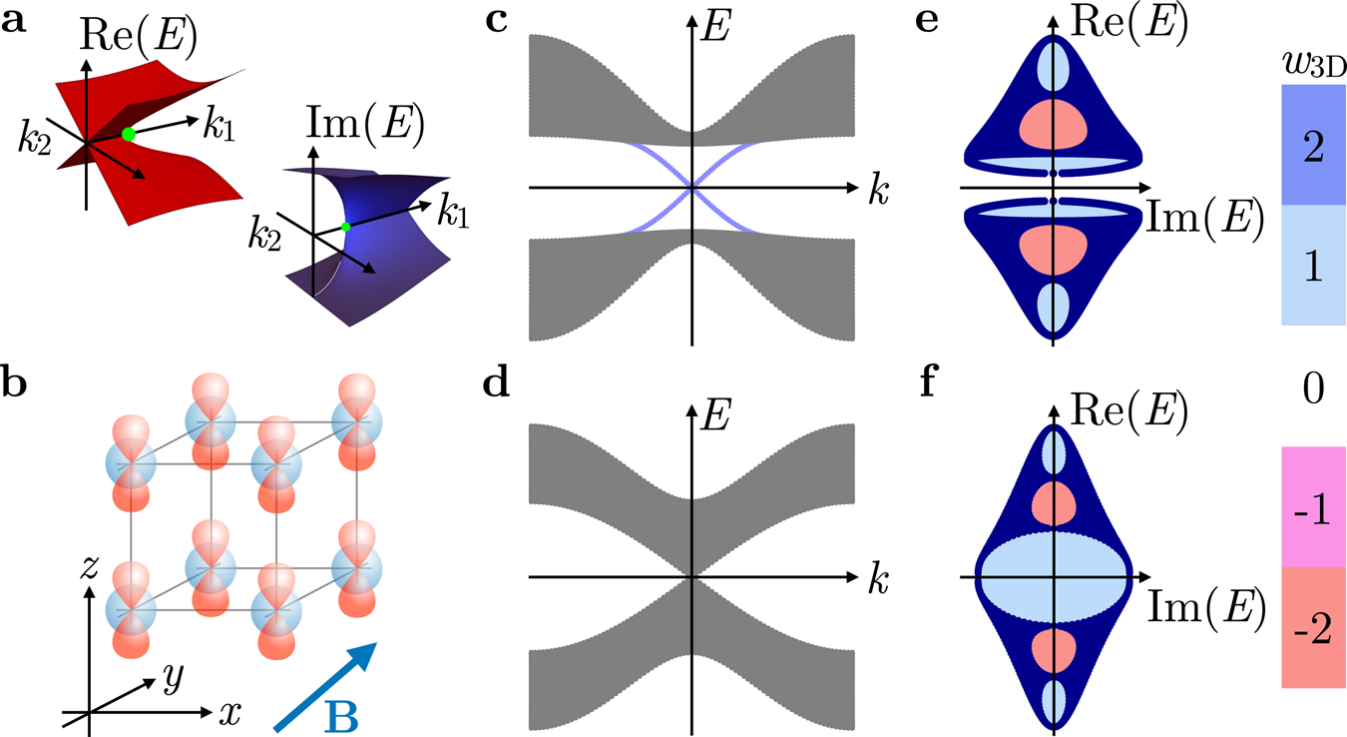
Constructing an ETI from a Hermitian 3D TI. **a** Schematic band structure near an exceptional point (green). **b** Real-space cubic lattice model of the 3D TI with *s* (*p*) orbitals depicted in blue (red). **c** Bulk spectrum of the Hermitian 3D TI in the nontrivial phase (1 < *M* < 3) along one momentum direction *k* with a superimposed Dirac surface state (blue). **d** Bulk spectrum of the Hermitian 3D TI at the transition point (*M* = 3) along one momentum direction *k*. **e** Bulk spectrum in the complex plane [Re(*E*), Im(*E*)] of the non-Hermitian model in Eq. (1) under PBC for all momenta *k*_*x*_, *k*_*y*_, *k*_*z*_ at zero magnetic field (*B* = 0, *M* = 2.3, *λ* = 1, and *δ* = 0.5). As ∣*M* − 3∣ > *δ*, a line gap opens along the imaginary axis, with six hole regions with corresponding point-gap invariants *w*_3D_. **f** The bulk spectrum for *M* = 3 and *δ* = 1 for the non-Hermitian model shows five hole regions, with corresponding point-gap invariants *w*_3D_.

Most of such meta-material platforms are accidentally or tunably lossy, such that their effective Hamiltonian description involves non-Hermitian terms due to the lack of energy conservation^10^. The same holds for interacting electronic quantum systems in which quasiparticles attain a finite lifetime, as manifested in a complex self-energy^11–14^. Starting from the initial classification of topological matter based on Hermitian Hamiltonians, the study of systems with non-negligible loss and gain calls for an extension to non-Hermitian topological matter. At this early stage of the field, several principles have been uncovered: (i) non-Hermitian systems have stable band degeneracies in two dimensions (2D), called exceptional points^15–17^ (Fig. 1a). (ii) Two different types of gaps have to be distinguished when eigenvalues are complex—line gaps, which can be adiabatically transformed into a Hermitian system, and point gaps, where this is not the case^18^. (iii) The topological bulk-boundary correspondence may break down for non-Hermitian systems due to the skin effect, which leads to dramatic shifts in the spectrum for open versus periodic boundary conditions (PBCs) as well as to a piling up of bulk states at the boundary^19–26^. (iv) The structure of topological invariants becomes more intricate, as complex-valued energy eigenvalues can themselves acquire a winding number^27,28^.

The key property of the single Dirac electron on the 3D TI surface is that it represents an anomaly: in purely 2D such a state can neither be regularized on a lattice (implied by the fermion doubling theorem) nor in the continuum (implied by gauge symmetry)^29,30^. Our search for a non-Hermitian analog of the 3D TI thus adopts a perspective of reverse-engineering: what could the anomalous non-Hermitian surface states be which necessitate a 3D topological bulk embedding? Two options come to mind: (1) a 2D band structure with a single exceptional point^31^; and (2) a single band with eigenvalues *E*(*k*_*x*_, *k*_*y*_) = *k*_*x*_ + i*k*_*y*_, which represents a vortex^32^, without the otherwise required antivortex. In this work, we introduce exceptional topological insulators (ETIs) as the paradigmatic class of 3D non-Hermitian topological systems. We here extend the notion of “insulator” to the case of point-gapped non-Hermitian systems, irrespective of their transport properties. Under PBCs, ETIs have a point gap in the spectrum, while in the presence of a boundary they support one of the above two types of surface states. We show that surface manipulations can interpolate between the cases (1) and (2). In particular, we demonstrate that our ETI models do not exhibit a non-Hermitian skin effect, such that the surface states are not overshadowed by a collapse of the point gap. In contrast to the conventional 3D TI, the surface states of an ETI do not require time-reversal symmetry for their protection and may therefore generically occur in non-Hermitian systems.

## Results

### Model

We formulate a microscopic electronic quantum model for an ETI. Our results, however, hold independently from this setting and readily carry over to systems with other degrees of freedom.

Consider a tight-binding model on a cubic lattice with an *s* and a *p* orbital at each site, each of which can be filled with spin *↑* and *↓* electrons (Fig. 1b). Let the Pauli matrices *σ*_*μ*_ and *τ*_*μ*_ act on the spin and orbital degrees of freedom, respectively, with *μ* = 0, *x*, *y*, *z*, and the 0th Pauli matrix as the 2 × 2 identity matrix. The Bloch Hamiltonian is defined as

1 $$\begin{array}{rcl}H({{{{{{{\bf{k}}}}}}}})&=&\left(\mathop{\sum}\limits_{j=x,y,z}\,\,\,\cos {k}_{j}-M\right)\,\,{\tau }_{z}{\sigma }_{0}+\lambda \,\,\,\mathop{\sum}\limits_{j=x,y,z}\sin {k}_{j}\ {\tau }_{x}{\sigma }_{j}\\ &&+\ [\sin \alpha \ {\tau }_{0}+\cos \alpha \ {\tau }_{z}]({{{{{{{\bf{B}}}}}}}}\cdot {{{{{{{\boldsymbol{\sigma }}}}}}}})+{{{{{{{\rm{i}}}}}}}}\delta \ {\tau }_{x}{\sigma }_{0}.\end{array}$$

For **B** = *δ* = 0, *H*(**k**) is a well-known Hamiltonian of a conventional 3D TI if 1 < ∣*M*∣ < 3 with phase transitions towards trivial insulators at ∣*M*∣ = 1 and ∣*M*∣ = 3 (see Fig. 1c). The parameter *M* controls the band inversion between *s* and *p* orbitals, while *λ* represents the spin-orbit coupling. Furthermore, **B** represents a Zeeman field, which we take to be **B** = (*B*, *B*, *B*)^⊤^ throughout, and *α* accounts for a possible imbalance between the *g*-factors of the *s* and *p* orbitals (for *α* = *π*/2 the *g*-factors are the same, for *α* = 0 they have opposite sign). The term proportional to *δ* introduces the non-Hermiticity. We provide a physical motivation for its specific form below.

Our regime of interest is close to the phase transition between the topological and trivial insulator phase (Fig. 1d), where the low-energy physics of the model is described by a 3D Dirac equation for *δ* = *B* = 0. For concreteness, we choose parameters *M* = 3 and *λ* = 1 throughout and then consider a finite *δ* (see Supplementary Note 2 for a full phase diagram). Assuming PBCs, we observe that *δ* opens a series of point gaps in the bulk complex spectrum of *H*(**k**) (Fig. 1e), while the line gap pertaining to the Hermitian 3D TI phase is closed for ∣*M* − 3∣ < *δ* (Fig. 1f). We posit that the constructed point-gapped Hamiltonian exhibits the phenomenology of an ETI in the presence of open boundary conditions (OBCs). The role of *B* will become clear once we study its surface states below; for now, we only require *B* to be small enough to not close the point gap at zero energy.

### Topological invariants

Information about the spectrum with OBC can be inferred from topological invariants computed from bulk states with PBC. While the palette of available topological invariants depends on the symmetry^18^, here we only consider point-gap invariants that remain in the absence of symmetry.

Specifically, in 3D there are three non-Hermitian weak integer winding numbers *w*_1D,*j*_ tied to specific directions in momentum space^33^. In addition, there is an intrinsically 3D integer invariant^18,28,34^ *w*_3D_ (see Methods). While non-vanishing *w*_1D,*j*_ have been related to the collapse of the point gap under OBC (non-Hermitian skin effect)^27,33^, the physical significance of *w*_3D_ has previously not been clarified.

In the following, we demonstrate the bulk-boundary correspondence for a system with nonzero *w*_3D_ that does not suffer from a skin effect and find that it exactly corresponds to the ETI as characterized above. The necessary condition for the absence of the skin effect, *w*_1D,*x*_ = *w*_1D,*y*_ = *w*_1D,*z*_ = 0, holds for the Hamiltonian in Eq. (1). The values of *w*_3D_ in the point gaps of the Hamiltonian are indicated in Fig. 1, e, f.

### Surface states

We study model (1) in the presence of an open boundary. Owing to the Zeeman term, the model is symmetric under 2*π*/3-rotations around the (111)-axis, implying the OBC spectra for *x*, *y*, and *z* termination are equivalent. We thus consider OBC with *N* layers in *z* direction and PBC in *x* and *y* directions (the “slab geometry”). We set *δ* = *λ* = 1 and *M* = 3 throughout the discussion.

We begin with the case *B* = 0, when the problem is analytically tractable^35^ at *k*_*x*_ = *k*_*y*_ = 0 (see Supplementary Note 1 and 2), and the characteristic polynomial ${E}^{4}{({E}^{2}-1)}^{2(N-1)}$ has four roots at *E* = 0. However, we find only two linearly independent eigenstates at this energy (one localized at either surface), indicating that the Hamiltonian is defective at this point. Solving for the dispersion of the zero-energy states perturbatively in $k={({k}_{x}^{2}+{k}_{y}^{2})}^{1/2}$, we find ${E}_{N}({k}_{x},{k}_{y})=\pm \sqrt{\pm {{{{{{{\rm{i}}}}}}}}}\ {2}^{N/2}\sqrt{k}$. In the thermodynamic limit *N* → *∞*, this leads to an infinitely steep set of eigenvalue branches, which is why we call *k*_*x*_ = *k*_*y*_ = 0 an infernal point. A similar exceptional point with an order equivalent to system size was found in ref. ^35^, although using a two-band model (see also Supplementary Note 6).

We argue however that the infernal point exhibits a fine-tuned, rather than the generic, surface-state structure of an ETI. Specifically, we find the surface spectrum to be regularized by small perturbations, such as the finite Zeeman term in Eq. (1), which we expect to be generally present in a physical realization. Note that *B* ≠ 0 breaks the isotropy of the model by selecting the (1,1,1)-direction. While the OBC spectra in the * x*, *y*, and *z* direction are equivalent, a surface termination perpendicular to the (1,1,1)-direction still exhibits the infernal point. We first study the effect of the Zeeman term with *α* = 0 and *α* = *π*/2 separately and then discuss the transition between the two.

**Fig. 2:**
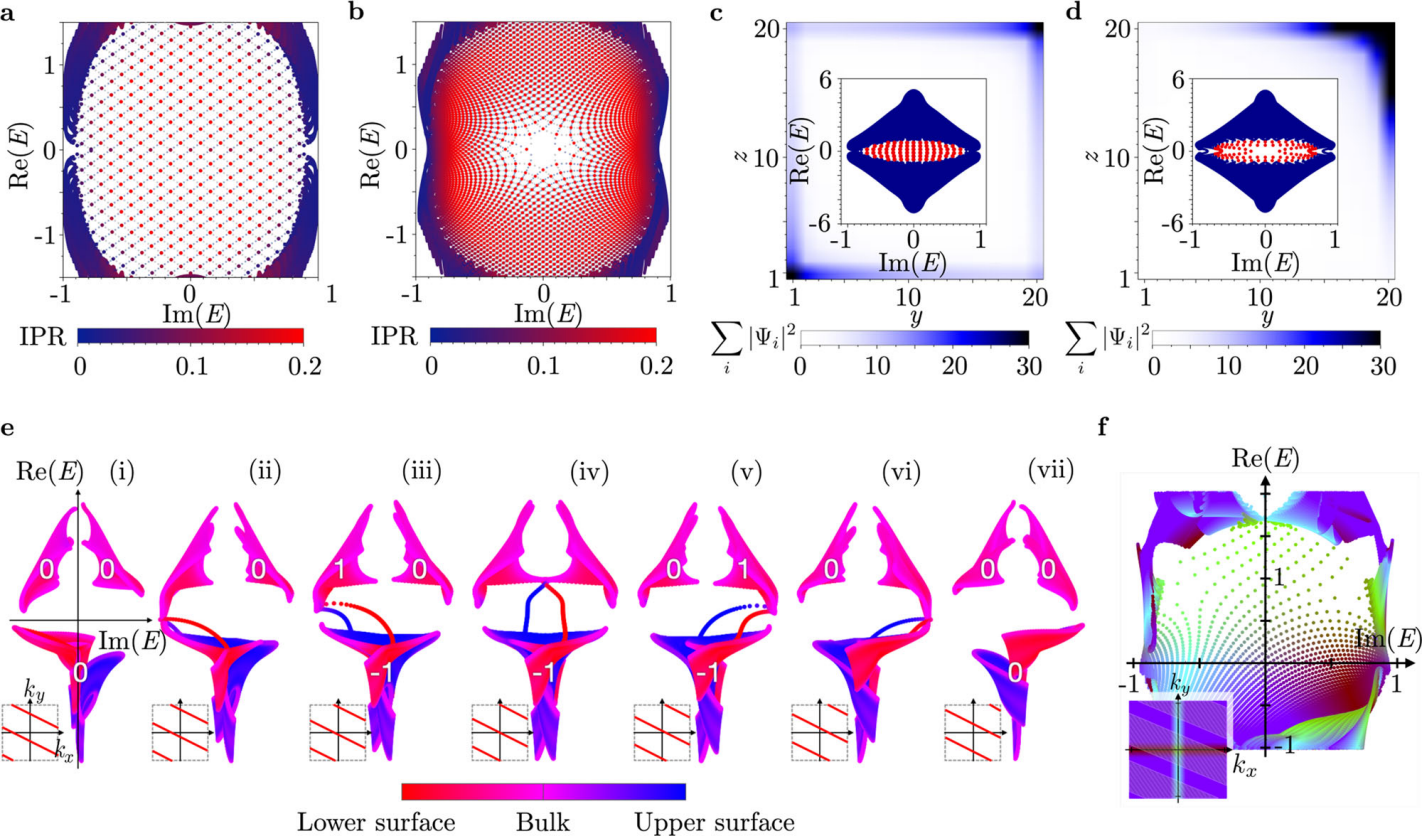
Open boundary condition (OBC) spectra of the exceptional topological insulator. **a**, **b** Complex-energy spectrum for the central point gap [cf. Fig. 1f] for OBC in the * z* direction and for a momentum resolution of *δk* = 2*π*/800 in both *k*_*x*,*y*_-directions (*B* = 0.2, *δ* = 1). Red tones highlight states of large surface localization, with the light gray mesh of finer resolution *δk* = 2*π*/2400 indicating the band energies along lines parallel to either the *k*_*x*_ or *k*_*y*_ axis. Concretely, we compute the inverse participation ratio (IPR), which sums the wavefunction over the lattice sites along the finite dimension *z*. A completely delocalized (bulk) state corresponds to vanishing IPR. A single surface band covers the point gap with a regular grid for *α* = *π*/2 (**a**), whereas the presence of an exceptional point at the origin for *α* = 0 is revealed by a 2*π* disclination (**b**) (see also Supplementary Note 3). **c**, **d** Summed density profile ∑_*i*_∣*ψ*_*i*_∣^2^ of the eigenstates in the central point gap for open boundaries in *z* and *y* direction indicated with color scale (*B* = 0.2, *δ* = 1), showing a surface skin effect. The insets display the corresponding energy spectrum in the complex plane, with the selected states highlighted in red. The single surface band for *α* = *π*/2 shows a boundary localization in two opposite corners (**c**), whereas the exceptional point for *α* = 0 localizes all surface states in one corner (**d**). **e** Chern number (white numbers) evolution for a series of cuts in the surface BZ to illustrate the chiral charge pumping around the point gap for *α* = 0.9 (*B* = 0.5, *δ* = 1). The employed colormap highlights the localization of the respective eigenstates. **f** Combination of cuts from panel **e**, zoomed onto the central point gap, highlighting how the single-sheet covering in panels **a**, **b** relates to the chiral edge states in **e**. The inset indicates the employed color scheme for momenta in the surface BZ as well as the cuts used for diagonalization (shaded area). Only bulk states and states localized on the upper surface are displayed (*α* = 0.9, *B* = 0.5, *δ* = 1).

For *α* = *π*/2 (Fig. 2a), we find a single sheet of complex-eigenvalue states localized on the top surface to cover the point-gap region in the complex-energy plane (along with another sheet with eigenstates localized on the bottom surface). We numerically determine the “dispersion” of the surface state as *E*(*k*_*x*_, *k*_*y*_) ∝ (*k*_*x*_ + i*k*_*y*_) to linear order in *k*_*x*_ and *k*_*y*_. Thus, the system has a single Fermi point in the surface Brillouin zone (BZ) at *k*_*x*_ = *k*_*y*_ = 0.

An odd number of Fermi points is impossible in a strictly 2D model due to the non-Hermitian fermion doubling theorem^31^. This follows because eigenvalues of a 2D Hamiltonian define continuous maps from a 2D BZ torus to the complex plane ${\mathbb{C}}$. Since the BZ has no boundary, each point in ${\mathbb{C}}$, including *E* = 0, must be the image of an even number of momenta in the BZ. The single-sheet covering of the point gap exhibited by the ETI is thus anomalous and only possible because it is connected to a 3D bulk spectrum.

For *α* = 0 (Fig. 2b), a single exceptional point is found on the surface of the ETI. Likewise, this situation is anomalous since also an odd number of exceptional points cannot be realized in purely 2D according to the non-Hermitian fermion doubling theorem^31^. In fact, each energy in the point-gap region is covered exactly once, reminiscent of the previously discussed *α* = *π*/2 case.

Interpolating between *α* = 0 and *α* = *π*/2 changes the surface spectrum by moving the exceptional point on the surface out of the point gap into the spectral region of the bulk states. This is analogous to surface states of a conventional 3D TI, where the topological surface Dirac cone can either be found within the bulk energy gap or can be “buried” in the bulk energy bands^3,4^, leaving a single band sheet on the surface (see Supplementary Note 3). We find an exact bulk-boundary correspondence between invariant *w*_3D_ and the number of point-gap covering surface-state sheets by considering a Hermitian doubled Hamiltonian^25^ that corresponds to two ETI copies related by Hermitian conjugation (see Methods).

Another generic property of the ETI emerges when considering OBC in two directions (while keeping PBC in the third direction), where a surface skin effect^36^ localizes order *N* states exponentially at the hinges. This is related to a higher order skin effect^37^. One particular difference between the two values of *α* can be seen in the localization of the modes, for *α* = *π*/2, the spectral weight of the surface states concentrates on two opposite corners (Fig. 2c). By contrast, for *α* = 0 the surface exceptional point localizes all the surface states exponentially towards one side (Fig. 2d).

### Berry flux

The anomalous boundary states of an ETI are tied to the nontrivial bulk topological invariant (5) via the bulk-boundary correspondence. To substantiate this claim we reformulate the invariant as follows. Recall that a non-Hermitian Hamiltonian with a point gap can be continuously deformed into a unitary matrix while preserving locality and band topology^28^. Such a deformed Hamiltonian has orthogonal eigenstates with eigenvalues lying on a unit circle, *E*(**k**) → *e*^i*ε*(**k**)^, suggesting an interpretation as a periodically driven (i.e., “Floquet”) quantum system^38^. In this context, *ε* is referred to as quasi-energy. Then (see Supplementary Note 4 or ref. ^38^),

2 $${w}_{{{{{{{{\rm{3D}}}}}}}}}={C}_{{{{{{{{\rm{FS}}}}}}}}(\mu )},$$

where the right-hand side denotes the Chern number of the Fermi surface at (arbitrary) quasi-energy *μ*, i.e., FS(*μ*) = {**k** ∈ BZ ∣ ∃ *a*: *ε*^*a*^(**k**) = *μ*} with *a* the band index. Equation (2) allows to interpret a nonzero *w*_3D_ as counting the quanta of Berry flux that circulate around the point gap in the complex-energy plane^39,40^.

The Berry flux can be related to the ETI surface states as follows. Consider the system with OBC in *z* direction and PBC otherwise and assume a series of cuts through the 2D surface BZ. Each cut represents a fictitious 2D non-Hermitian system with OBC in one direction. In 2D, isolated bands can be characterized by an integer Chern number *C*^17,42^. If ∣*C*∣ is nonzero, the corresponding number of edge-localized topological modes connect the band with the other bands inside the complex plane, similar to Hermitian Chern insulators that describe the integer quantum Hall effect in lattice systems^43^.

We visualize the construction for model (1) in Fig. 2e along the cuts shown in the insets. We observe that the four bands of the model project to three distinct regions in the complex plane. Panels (e-ii), (e-iv), and (e-vi) indicate critical cuts when the bands exchange Chern numbers, while in the intermediate regions the bands exhibit fixed values *C* = 0 or *C* = ± 1. We observe in the sequence of cuts that a Chern dipole is formed on the left side of the point gap, by transferring a Chern number +1 (in a clockwise direction) between the two bands that touch in the second panel. The topological charge *C* = +1 is then transferred between the upper left and the upper right band, and finally back to the lower band. Thus, a quantum +1 of Berry flux is pumped clockwise around the point gap, in accordance with the rewriting of *w*_3D_ in Eq. (2). Note that Fig. 2e also indicates the boundary-localized edge modes which at each stage connect bands with opposite Chern numbers. As such, they necessarily swipe over the entire point-gap region during the pumping process, thus forming the protected topological surface state of the ETI. Consequently, the Chern number flow around the point gap corresponds to a rotation of the chiral edge state, which leads to the anomalous net surface chirality. Combining the series of cuts with a momentum resolution in Fig. 2f provides a visual connection between the numerically obtained surface spectra and the topological invariant.

**Fig. 3:**
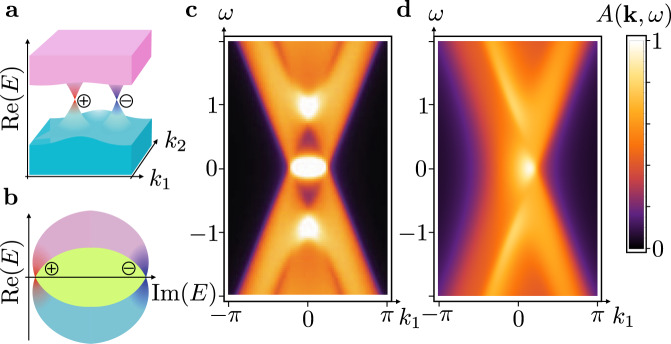
Inducing an ETI in a Hermitian Weyl semimetal. **a** Schematic band structure of a Weyl semimetal with two Weyl cones of opposite chiral charge (red vs. blue) connecting bulk valence (cyan) and conduction (pink) bands. **b** An ETI with a point gap (green) arises if we assign different lifetimes to the two chiral fermions. The bulk states carry a quantum of Berry flux around the point gap. **c**, **d** Spectral density derived from the surface Green’s function^41^ *G*(**k**, *ω*) via $A({{{{{{{\bf{k}}}}}}}},\omega )=-1/\pi \ {{{{{{{\rm{Im}}}}}}}}\ {{{{{{{\rm{Tr}}}}}}}}\ G({{{{{{{\bf{k}}}}}}}},\omega +{{{{{{{\rm{i}}}}}}}}\epsilon )$, along momenta *k*_1,2_ = (*k*_*x*_ ± *k*_*y*_)/2 and for frequency *ω*. We set the smearing factor to *ϵ* = 0.1 and the non-Hermitian term to *δ* = 0 (**c**) resp. to *δ* = −1/2 (**d**), for Hamiltonian parameters *M* = 3, *α* = *π*/2, and *B* = 1/2. We added the term i*δτ*_0_*σ*_0_ to the Hamiltonian in order for all eigenvalues to have a nonpositive imaginary part.

Based on the relation between *w*_3D_ and the Berry flux, we expect ETIs to arise generically out of critical 3D band structures, in particular from Weyl semimetals^44^. Note that for *δ* = 0 and *α* = *π*/2, Hamiltonian (1) is precisely a Weyl semimetal with a pair of Weyl points at **k** = ±**B**. These are a source and a sink of unit Berry flux^45^. With OBC, the Weyl points are connected by surface Fermi arcs. Upon including *δ* ≠ 0 the eigenvalues of the Weyl points acquire different imaginary parts, such that inevitably a bulk spectrum with a point gap emerges (Fig. 3a and b). Under OBC, the point gap is filled with Fermi-arc states that connect the Weyl points (green). These are the ETI surface states. In Fig. 3c and d we show the surface spectral function for a Weyl semimetal and an ETI. The latter is characterized by only one visible Weyl cone and a broad signal from the surface states that gets sharper closer to the cone. This indicates that the ETI, which is a point-gapped non-Hermitian topological phase, is spectroscopically a semimetal with a vanishing density of states at ${{{{{{{\rm{Re}}}}}}}}(E)=0$.

### Non-Hermitian terms

We finish by discussing how the non-Hermitian term i*δτ*_*x*_*σ*_0_ may arise in a 3D TI and which measurable consequences it has. In principle, a tailored orbital-dependent coupling to a lossy mode would suffice to give rise to such a term. For instance, we can consider an additional impurity, e.g., *f*-orbital at energy *μ*_f_ in the unit cell with no dispersion but finite lifetime Γ as a representative source for non-Hermitian contributions to the electronic self-energy. If this short-lived *f*-electron couples to the *s*, *p* orbitals of the topological insulator with hopping strength *t*_f_, the single-electron Green’s function acquires a complex self-energy ${{\Sigma }}={{{{{{{\rm{i}}}}}}}}{t}_{{{{{{{{\rm{f}}}}}}}}}^{2}/({{\Gamma }}-{{{{{{{\rm{i}}}}}}}}{\mu }_{{{{{{{{\rm{f}}}}}}}}})({\tau }_{0}{\sigma }_{0}+{\tau }_{x}{\sigma }_{0})$ (see Supplementary Note 5). For *μ*_f_ ≪ Γ, the non-Hermitian term dominates and, up to an overall imaginary shift in the spectrum, contributes the desired non-Hermitian term in Eq. (1). Alternatively, electron–phonon-scattering can act as a source for the non-Hermitian terms in the self-energy, likewise leading to the topological features of an ETI.

Classical analogs to quantum mechanical topological states can be constructed in a variety of platforms, including phononic^46,47^, photonic^48–50^, and electrical^51^ meta-materials. The ETI is no exception to this. For realizations by design, a two-band model^35^, which, however, requires a more complicated anti-Hermitian term than the four-band model of Eq. (1), may be more amenable (see Supplementary Note 6).

## Discussion

We introduced 3D ETIs, a phase of matter governed by a local non-Hermitian (Hamiltonian) operator with a point gap and topological surface states. In particular, we show that a Weyl semimetal with two Weyl nodes at the Fermi energy generically becomes an ETI under a non-Hermitian perturbation that opens a point gap. Besides the realization of ETIs in (meta-)materials, open questions for future research include: How are ETI phases further differentiated by the addition of symmetries? What are alternative representations of the topological invariants in terms of symmetry indicators? What role do interactions play in the stability of ETIs? Our findings are the first step towards a microscopic understanding of such non-Hermitian topological matter.

## Methods

### ETI tight-binding model

As an illustrative model for the ETI phase, we consider in the main text a cubic lattice with two orbitals *s* (*γ* = 0) and *p* (*γ* = 1) and spin *↑*, *↓* per site. The lattice is spanned by the unit vectors **e**_*i*_, *i* = *x*, *y*, *z*, giving rise to the tight-binding Hamiltonian

3 $$H	= -M\mathop{\sum}\limits_{{{{{{{{\bf{r}}}}}}}},\gamma }{(-1)}^{\gamma }\ {c}_{{{{{{{{\bf{r}}}}}}}},\gamma }^{{{{\dagger}}} }{\sigma }_{0}{c}_{{{{{{{{\bf{r}}}}}}}},\gamma }\\ 	\quad+\frac{1}{2}\mathop{\sum}\limits_{{{{{{{{\bf{r}}}}}}}},\gamma }\mathop{\sum}\limits_{i=x,y,z}{(-1)}^{\gamma }\ {c}_{{{{{{{{\bf{r}}}}}}}}+{{{{{{{{\bf{e}}}}}}}}}_{i},\gamma }^{{{{\dagger}}} }{\sigma }_{0}{c}_{{{{{{{{\bf{r}}}}}}}},\gamma }+{{{{{{{\rm{h.c.}}}}}}}}\\ 	\quad +\frac{\lambda }{2{{{{{{{\rm{i}}}}}}}}}\mathop{\sum}\limits_{{{{{{{{\bf{r}}}}}}}},\gamma }\mathop{\sum}\limits_{i=x,y,z}\ {c}_{{{{{{{{\bf{r}}}}}}}}+{{{{{{{{\bf{e}}}}}}}}}_{i},\gamma +1}^{{{{\dagger}}} }{\sigma }_{i}{c}_{{{{{{{{\bf{r}}}}}}}},\gamma }+{{{{{{{\rm{h.c.}}}}}}}}\\ 	\quad +\mathop{\sum}\limits_{{{{{{{{\bf{r}}}}}}}},\gamma }\mathop{\sum}\limits_{i=x,y,z}{B}_{i}\left[{(-1)}^{\gamma }\cos (\alpha )+\sin (\alpha )\right]\ {c}_{{{{{{{{\bf{r}}}}}}}},\gamma }^{{{{\dagger}}} }{\sigma }_{i}{c}_{{{{{{{{\bf{r}}}}}}}},\gamma }\\ 	\quad +{{{{{{{\rm{i}}}}}}}}\delta \mathop{\sum}\limits_{{{{{{{{\bf{r}}}}}}}},\gamma }\ {c}_{{{{{{{{\bf{r}}}}}}}},\gamma +1}^{{{{\dagger}}} }{\sigma }_{0}{c}_{{{{{{{{\bf{r}}}}}}}},\gamma },$$

where we use *γ* modulo 2 and the Pauli matrices as *σ*_*μ*_, *μ* = 0, *x*, *y*, *z* with the 0th Pauli matrix as the 2 × 2 identity. The operator ${c}_{{{{{{{{\bf{r}}}}}}}},\gamma }^{{{{\dagger}}} }=\left({c}_{{{{{{{{\bf{r}}}}}}}},\gamma ,\uparrow }^{{{{\dagger}}} }\ {c}_{{{{{{{{\bf{r}}}}}}}},\gamma ,\downarrow }^{{{{\dagger}}} }\right)$ then creates an electron in orbital *γ* at lattice site **r** with the respective spin orientation.

### Topological invariants

Two important invariants exist for 3D non-Hermitian systems in the absence of any symmetry. First, specific directions in momentum space are equipped with a weak integer invariant^33^,

4 $${w}_{{{{{{{{\rm{1D}}}}}}}},j}=-{{{{{{{\rm{i}}}}}}}}\int_{{{{{{{{\rm{BZ}}}}}}}}}\frac{{d}^{3}{{{{{{{\bf{k}}}}}}}}}{{(2\pi )}^{3}}{{{{{{{\rm{Tr}}}}}}}}[{Q}_{j}({{{{{{{\bf{k}}}}}}}})],$$

with ${Q}_{j}({{{{{{{\bf{k}}}}}}}})={[H({{{{{{{\bf{k}}}}}}}})-E]}^{-1}{\partial }_{{k}_{j}}[H({{{{{{{\bf{k}}}}}}}})-E]$, *E* is any complex value in the point gap, *j* = *x*, *y*, *z*, and BZ = [−*π*, *π*]^3^ denotes the 3D Brillouin zone. A nonzero *w*_1D,*j*_ indicates the non-Hermitian skin effect^27,33^, under which the spectrum collapses upon considering OBCs. Hence, a vanishing *w*_1D,*j*_ = 0 is required for the observation of topological surface states.

Such a scenario is indicated by an intrinsically 3D integer invariant^18,28,34^

5 $${w}_{{{{{{{{\rm{3D}}}}}}}}}=-{\int}_{{{{{{\!\!{{\rm{BZ}}}}}}}}}\frac{{d}^{3}{{{{{{{\bf{k}}}}}}}}}{24{\pi }^{2}}{\epsilon }_{ijk}{{{{{{{\rm{Tr}}}}}}}}[{Q}_{i}({{{{{{{\bf{k}}}}}}}}){Q}_{j}({{{{{{{\bf{k}}}}}}}}){Q}_{k}({{{{{{{\bf{k}}}}}}}})],$$

where the summation of repeated indices *i*, *j*, *k* is implied and *ϵ*_*ijk*_ is the Levi-Civita symbol. The physical significance of a nonzero *w*_3D_ is the ETI phase presented in this manuscript.

### Proof of bulk-boundary correspondence

The ETI bulk-boundary correspondence builds on the fact that the point gap of an ETI necessarily fills up with surface-localized states when OBCs are introduced. These midgap surface states are topologically protected by the nonzero ETI bulk invariant *w*_3D_ without the assumption of additional symmetries, similarly to how the edge states of a Chern insulator are protected by a nonzero Chern number.

We consider an ETI that is described by a Bloch Hamiltonian *Q*(**k**) with a point gap around the complex-energy *E*_0_, and a winding number ∣*w*_3D_∣ = 1. Since the winding number is quantized, we may equivalently consider any other *E*_0_ lying within the same point gap. We now define the Hermitian double

6 $$\tilde{H}(Q({{{{{{{\bf{k}}}}}}}})-{E}_{0})=\left[\begin{array}{cc}0&Q({{{{{{{\bf{k}}}}}}}})-{E}_{0}\\ Q{({{{{{{{\bf{k}}}}}}}})}^{{{{\dagger}}} }-{E}_{0}^{* }&0\end{array}\right],$$

which describes a topological insulator in Altland–Zirnbauer class AIII with a single surface Dirac cone^52^. Correspondingly, the Hamiltonian ${\tilde{H}}_{{{\mbox{slab}}}}$ obtained by placing the real-space version of $\tilde{H}({{{{{{{\bf{k}}}}}}}})$ in a slab geometry satisfies $\det ({\tilde{H}}_{{{\mbox{slab}}}})=0$: for both surfaces, the chiral symmetry of ${\tilde{H}}_{{{\mbox{slab}}}}$ enforces a spectral pinning of the surface Dirac crossing to zero energy. We do not resolve any remaining momentum quantum numbers because a surface Dirac cone in class AIII is not pinned to any particular surface momentum. In the slab geometry, the decomposition reads ${\tilde{H}}_{{{\mbox{slab}}}}({Q}_{{{\mbox{slab}}}}-{E}_{0})$, where *Q*_slab_ is the non-Hermitian Hamiltonian obtained by placing the real-space version of *Q*(**k**) in the slab geometry. The surface Dirac cones of ${\tilde{H}}_{{{\mbox{slab}}}}$ then imply

7 $$\det ({\tilde{H}}_{{{\mbox{slab}}}})= \, \det [-({Q}_{{{\mbox{slab}}}}-{E}_{0})({Q}_{{{\mbox{slab}}}\,}^{{{{\dagger}}} }-{E}_{0}^{* })]=0\\ \, \to\; \det ({Q}_{{{\mbox{slab}}}}-{E}_{0})=0,$$

from which we deduce that *Q*_slab_ has at least one eigenvalue equal to *E*_0_.

After establishing the presence of protected midgap states in the slab spectrum of an ETI, described by a Bloch Hamiltonian *Q*(**k**), we next derive its unique topological surface characteristic: the surface chirality. By this, we mean the accumulated winding of all energies of the states on a given surface around a reference energy *E*_0_ (chosen to lie within the point gap), which can be calculated via the formula

8 $$\nu ({E}_{0})=\frac{1}{2\pi }\oint_{\gamma ({E}_{0})}{\partial }_{k}\left\{\mathop{\sum}\limits_{i}{{{{{{{\rm{Arg}}}}}}}}[{E}_{i}({{{{{{{{\bf{k}}}}}}}}}_{\perp })-{E}_{0}]\right\}{{{{{{{\rm{d}}}}}}}}k\in {\mathbb{Z}},$$

where the path of momenta *γ*(*E*_0_) is obtained as the surface Brillouin zone preimage of any connected set of energies *E*_*i*_[*γ*(*E*_0_)] that encircles *E*_0_ counter-clockwise in the complex plane. Note that all *E*_*i*_[*γ*(*E*_0_)] should lie within the point gap. We will prove that ∣*ν*(*E*_0_)∣ = 1 is nonzero for all choices of *E*_0_.

Recall the expression for the surface winding number of a Hermitian topological insulator in class AIII^53^,

9 $${\nu }_{{{{{{{{\rm{Hermitian}}}}}}}}}=\frac{1}{2\pi }\oint _{\lambda }{{{{{{{\rm{Im}}}}}}}}\ {{{{{{{\rm{tr}}}}}}}}\left[q{\left({{{{{{{{\bf{k}}}}}}}}}_{\perp }\right)}^{-1}{\partial }_{k}q({{{{{{{{\bf{k}}}}}}}}}_{\perp })\right]{{{{{{{\rm{d}}}}}}}}k\in {\mathbb{Z}},$$

where *q*(**k**_⊥_) forms the Hermitian surface Hamiltonian ${\tilde{H}}_{{{\mbox{surface}}}}(q({{{{{{{{\bf{k}}}}}}}}}_{\perp }))$ and *λ* is any (possibly disconnected) path in the surface Brillouin zone that encloses all surface Dirac cones in the spectrum of ${\tilde{H}}_{{{\mbox{surface}}}}(q({{{{{{{{\bf{k}}}}}}}}}_{\perp }))$ counter-clockwise and only covers surface-localized states. ∣*ν*_Hermitian_∣ then counts the number of topologically protected surface Dirac cones.

We now relate the Hermitian surface winding number *ν*_Hermitian_ to the non-Hermitian surface chirality *ν*. In the absence of a collapse of the bulk spectrum of *Q*(**k**) as we open the boundary conditions, we can interpret [*q*(**k**_⊥_) − *E*_0_] as the effective surface Hamiltonian of the ETI [*Q*(**k**) − *E*_0_] [whose Hermitian double is $\tilde{H}(Q({{{{{{{\bf{k}}}}}}}})-{E}_{0})$ in Eq. (6)]. Also, as long as *E*_0_ lies in the point gap, the nontrivial ETI invariant ∣*w*_3D_∣ = 1 implies a single surface Dirac cone for the Hermitian double^52^, resulting in the equality

10 $${\pm 1}=	 \, {\nu }_{{{{{{{{\rm{Hermitian}}}}}}}}}({E}_{0})\\ =	 \, \frac{1}{2\pi }\oint_{\lambda ({E}_{0})}{\partial }_{k}{{{{{{{\rm{tr}}}}}}}}\left\{{{{{{{{\rm{Im}}}}}}}} \;{{{{{{\mathrm{log}}}}}}}\,[q({{{{{{{{\bf{k}}}}}}}}}_{\perp })-{E}_{0}]\right\}{{{{{{{\rm{d}}}}}}}}k\\ =	 \, \nu ({E}_{0}),$$

where we substituted *λ* → *λ*(*E*_0_) (the location of the surface Dirac cone varies with *E*_0_), and then identified *γ*(*E*_0_) = *λ*(*E*_0_). It remains to be shown that our definitions of *λ*(*E*_0_) and *γ*(*E*_0_) are compatible, that is, that the eigenvalues of *q*[*λ*(*E*_0_)] wind around *E*_0_. This must be so because *ν*(*E*_0_) could otherwise not take on nonzero values. Furthermore, since *ν*(*E*_0_) is quantized, any other choice of *γ*(*E*_0_) that has the abovementioned properties is equally valid, thus completing our proof.

In conclusion, we find that the surface band structure of an ETI is characterized by an anomalous net chirality, which cannot be realized in a purely 2D system, but is instead enabled by the presence of the topologically nontrivial 3D bulk.

## Supplementary information

Supplementary Information

## Data Availability

All information needed to evaluate the conclusions in the paper are present in the paper and/or the supplementary information. Additional data are available from the corresponding authors upon reasonable request.

## Code availability

The code used to generate the figures in this work is available upon reasonable request.
