# Supplementary material for: Exceptional topological insulators
Source: Nat Commun. 2021 Sep 28;12:5681. doi: 10.1038/s41467-021-25947-z (PMC8478920; doi:10.1038/s41467-021-25947-z)
Supplement: Supplementary file 1 — Supplementary Information [file 41467_2021_25947_MOESM1_ESM.pdf]

Supplementary Information: Exceptional Topological Insulators

M. Michael Denner,[1] Anastasiia Skurativska,[1] Frank Schindler,[1,2]
Mark H. Fischer,[1] Ronny Thomale,[3] Tomáš Bzdušek,[4,1] and Titus Neupert[1]

[1]*Department of Physics, University of Zurich, Winterthurerstrasse 190, 8057 Zurich, Switzerland*
[2]*Princeton Center for Theoretical Science, Princeton University, Princeton, NJ 08544, USA*
[3]*Institut für Theoretische Physik und Astrophysik, Universität Würzburg, 97074 Würzburg, Germany*
[4]*Condensed Matter Theory Group, Paul Scherrer Institute, 5232 Villigen PSI, Switzerland*

(Dated: September 7, 2021)

SUPPLEMENTARY NOTE 1: DIRAC THEORY FOR THE ETI

We here show how the infernal point of the Hamiltonian in Eq. (1) of the main text in presence of open boundary conditions can be derived from a Dirac equation approach, similar to how the gapless surface Dirac fermion of a Hermitian 3D TI can be obtained as a zeromode that is localized at domain walls of a 3D bulk Dirac mass. In comparison to the two zero-energy states of a 3D TI surface Dirac fermion, we find only a single zero-energy state for the ETI, underlining the fact that the ETI surface in some sense realizes "half" of a Hermitian Dirac cone (recall that upon the introduction of non-Hermitian terms, a 2D Dirac cone generically splits into two exceptional points).

We start with the Bloch Hamiltonian of a three-dimensional exceptional topological insulator (ETI), corresponding to Eq. (1) of the main text with the parameter choice $M = 3$, $\lambda = 1$, $\mathbf{B} = \mathbf{0}$, giving

$$H(\mathbf{k}) = \left(\sum_{i=x,y,z} \cos k_i - 3\right) \tau_z\sigma_0 + \sum_{i=x,y,z} \sin k_i \tau_x\sigma_i + \mathrm{i}\delta\tau_x\sigma_0, \tag{1}$$

which describes a four-fold Dirac crossing at $\mathbf{k} = (0, 0, 0)$ that is gapped out by the non-Hermitian term multiplying δ. Unless otherwise stated, we assume $\delta > 0$. $H(\mathbf{k})$ then has a nontrivial winding number[1] $w_{3\mathrm{D}} = 1$. The long-wavelength limit of the model is given by the Dirac theory

$$H_{\mathrm{D}}(\mathbf{k}) = \sum_{i=x,y,z} k_i\tau_x\sigma_i + \mathrm{i}\delta\tau_x\sigma_0. \tag{2}$$

We want to derive the band structure of the surface with normal in z-direction, where k_x and k_y remain as good momentum quantum numbers. For this, we model the interface between an ETI and the vacuum via the Dirac Hamiltonian

$$H_{\mathrm{D},z}(k_x, k_y) = \sum_{i=x,y} k_i\tau_x\sigma_i + (-\mathrm{i}\partial_z)\tau_x\sigma_z + \mathrm{i}\delta\theta(-z)\tau_x\sigma_0 - \mu\theta(z)\tau_z\sigma_0, \tag{3}$$

where $\mu > 0$ multiplies a Hermitian mass term that gaps Supplementary Eq. (1) at $\delta = 0$ into a trivial insulator and $\theta(z)$ is the Heaviside step function. We use the convention $\theta(0) = 0$.

Let us fix $k_x = k_y = 0$ for simplicity – we have numerically confirmed that the z-directed slab spectrum of the Bloch Hamiltonian (1) away from this point is gapped. Zero-energy states $\Psi(z)$ then satisfy

$$\begin{aligned}
&[(-\mathrm{i}\partial_z)\tau_x\sigma_z + \mathrm{i}\delta\theta(-z)\tau_x\sigma_0 - \mu\theta(z)\tau_z\sigma_0]\,\Psi(z) = 0,\\
&\quad\rightarrow\quad \tau_x\sigma_z\partial_z\Psi(z) = [\delta\theta(-z)\tau_x + \mathrm{i}\mu\theta(z)\tau_z]\,\sigma_0\Psi(z)\\
&\quad\rightarrow\quad \partial_z\Psi(z) = [\delta\theta(-z)\tau_0 + \mu\theta(z)\tau_y]\,\sigma_z\Psi(z)\\
&\quad\rightarrow\quad \partial_z\Psi_\lambda(z) = \lambda\,[\delta\theta(-z)\tau_0 + \mu\theta(z)\tau_y]\,\Psi_\lambda(z),
\end{aligned} \tag{4}$$

where we have chosen $\Psi_\lambda(z)$ as eigenstates of σ_z that satisfy $\sigma_z\Psi_\lambda(z) = \lambda\Psi_\lambda(z)$. We next solve the equation on both sides separately. For $z < 0$ we obtain

$$\partial_z\Psi^<_\lambda(z) = \lambda\delta\tau_0\Psi^<_\lambda(z) \quad\rightarrow\quad \Psi^<_{+,1}(z) = \mathcal{N}^< e^{\delta z}(1,0,0,0)^{\mathrm{T}}, \quad \Psi^<_{+,2}(z) = \mathcal{N}^< e^{\delta z}(0,0,1,0)^{\mathrm{T}}, \tag{5}$$

where $\mathcal{N}$ is a normalization factor, and the solutions corresponding to $\lambda = -1$ are not normalizable. For later use, let us note that the following linear combination is an equally valid zero-energy solution for $z < 0$:

$$\Psi_{+}^{<}(z) \equiv \frac{\Psi_{+,2}^{<}(z) + \mathrm{i}\Psi_{+,1}^{<}(z)}{\sqrt{2}} = \frac{\mathcal{N}^{<}}{\sqrt{2}} e^{\delta z}(\mathrm{i},0,1,0)^{\mathrm{T}}, \tag{6}$$

Likewise, for $z > 0$ we obtain

$$\partial_z \Psi_{\lambda}^{>}(z) = \lambda\mu\tau_y \Psi_{\lambda}^{>}(z) \quad \to \quad \Psi_{-}^{>}(z) = \mathcal{N}^{>} e^{-\mu z}(0,-\mathrm{i},0,1)^{\mathrm{T}}, \quad \Psi_{+}^{>}(z) = \mathcal{N}^{>} e^{-\mu z}(\mathrm{i},0,1,0)^{\mathrm{T}}, \tag{7}$$

where again the remaining solutions are not normalizable. To obtain a viable zero-energy wavefunction for the entire range of z, we need to match solutions across $z = 0$. For simplicity, let us set $\delta = \mu \equiv \Delta$. There is then only a single matchable solution, with wavefunction

$$\Psi(z) = \sqrt{\frac{\Delta}{2}} e^{-\Delta|z|}(\mathrm{i},0,1,0)^{\mathrm{T}}, \quad H_{\mathrm{D},z}(0,0)\Psi(z) = 0. \tag{8}$$

SUPPLEMENTARY NOTE 2: ANALYTICAL TREATMENT OF THE ETI TIGHT-BINDING MODEL

In this section we analyze the tight-binding model from Eq. (1) of the main text analytically in some detail. We set $B = 0$ throughout the discussion, i.e. we retain isotropy. While this causes certain instabilities in the numerical computation of the spectrum, it also makes the problem more amenable for an analytical treatment. The discussion is organized into subsections as follows. We begin in Supplementary Note 2.A. with rotating the model to a basis that explicitly reveals its sublattice symmetry. While not necessary to enable the $w_{3\mathrm{D}}$ invariant, the sublattice symmetry greatly simplifies the phase diagram of the model. In Supplementary Note 2.B. we write the analytical form of the Hamiltonian in slab geometry with open boundary conditions. By analyzing the constructed slab Hamiltonian we reveal the existence of the macroscopically defective infernal point at zero surface momentum. Finally, in Supplementary Note 2.C. we perform a perturbative expansion in the characteristic polynomial of the slab Hamiltonian to infer the dispersion of the surface states around the infernal point. We conclude with analyzing the convergence of our perturbative result by comparing to a numerical computation.

A. Phase diagram and the sublattice symmetry

We begin with the model in Eq. (1) with $B = 0$, but we rotate the Pauli matrices as $\tau_z \mapsto \tau_x \mapsto \tau_y \mapsto \tau_z$. This brings the Hamiltonian to the form

$$H(\mathbf{k}; M, \lambda, \delta) = \begin{pmatrix} 0 & 0 & f(\mathbf{k};M) - \mathrm{i}\lambda\sin k_z + \delta & -\mathrm{i}\lambda\sin k_x - \lambda\sin k_y \\ 0 & 0 & -\mathrm{i}\lambda\sin k_x + \lambda\sin k_y & f(\mathbf{k};M) + \mathrm{i}\lambda\sin k_z + \delta \\ f(\mathbf{k};M) + \mathrm{i}\lambda\sin k_z - \delta & \mathrm{i}\lambda\sin k_x + \lambda\sin k_y & 0 & 0 \\ \mathrm{i}\lambda\sin k_x - \lambda\sin k_y & f(\mathbf{k};M) - \mathrm{i}\lambda\sin k_z - \delta & 0 & 0 \end{pmatrix}, \tag{9}$$

where $f(\mathbf{k}; M) = \sum_{j=x,y,z} \cos k_j - M$. Note that the rotated basis reveals a previously hidden sublattice symmetry (which is broken for finite $\propto B$). We denote the lower-left (upper-right) block as h_{LL} ($h_{\mathrm{UR}}^{\dagger}$), where we dropped the dependence on parameters for brevity. Note that because of the non-Hermitian term $\propto \delta$ we have $h_{\mathrm{LL}} \neq h_{\mathrm{UR}}$. Note that the point gap of Hamiltonian H at $E = 0$, implies through $0 \neq \det H = \det h_{\mathrm{LL}} (\det h_{\mathrm{UR}})^*$ that each of the blocks respects the point gap too (the asterisk '*' indicates complex conjugation), meaning that both matrix blocks have a well-defined inverse.

Recall from Eq. (5) in the main text that the winding number $w_{3\mathrm{D}}$ for a point gap at $E = 0$ is determined from matrices $Q_i = H^{-1}\partial_i H$ where $\partial_i \equiv \partial_{k_i}$. It follows from the block-off-diagonal form of the Hamiltonian that H^{-1} is block-off-diagonal with $(h_{\mathrm{UR}}^{\dagger})^{-1}$ in the lower-left and h_{LL}^{-1} upper-right position, such that

$$Q_i = \begin{pmatrix} h_{\mathrm{LL}}^{-1}\partial_i h_{\mathrm{LL}} & \mathbb{0} \\ \mathbb{0} & (h_{\mathrm{UR}}^{\dagger})^{-1}\partial_i h_{\mathrm{UR}}^{\dagger} \end{pmatrix} \tag{10}$$

is block-diagonal. As a consequence, it is possible to write the integrand in Eq. (5) as a sum of two contributions, which can be through simple manipulations brought into the form

$$\mathrm{Tr}[Q_i Q_j Q_k] = \mathrm{Tr}[h_{\mathrm{LL},i} h_{\mathrm{LL},j} h_{\mathrm{LL},k}] - \mathrm{Tr}[h_{\mathrm{UR},i} h_{\mathrm{UR},j} h_{\mathrm{UR},k}], \tag{11}$$

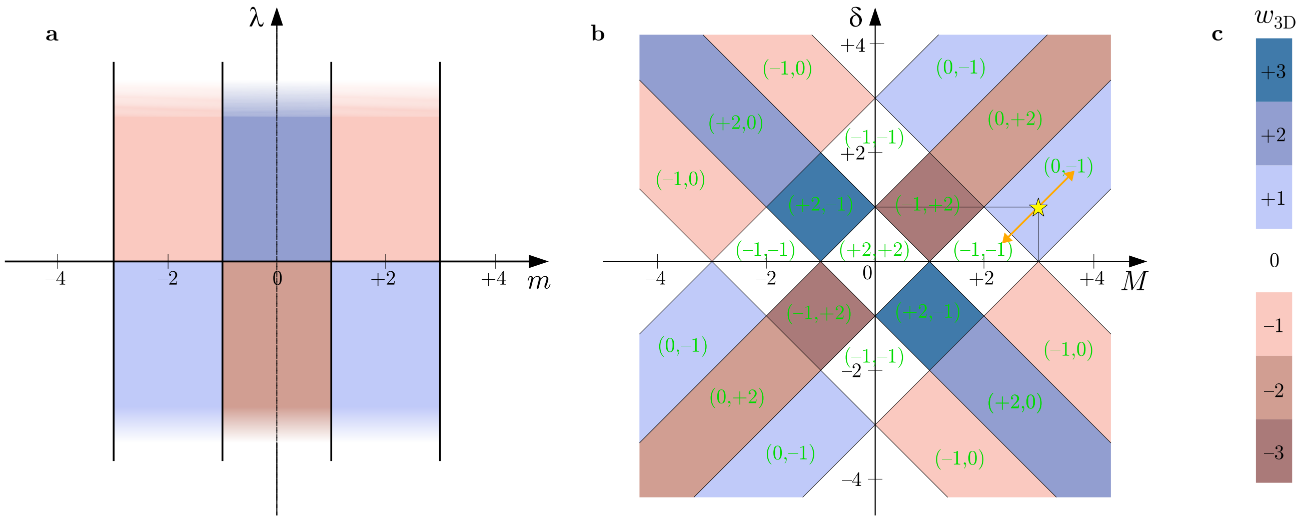

Supplementary Figure 1. **Phase diagrams for the ETI model.** **a** Winding number w_{3D} of the 2×2 block $h_0(\mathbf{k}; m, \lambda)$ in the (m, λ)-parameter space. **b** Phase diagram for the model in Eq. (1) of the main text for $B = 0$ and $\lambda > 0$ inside the (M, δ)-parameter plane. The pair of green numbers indicate the two invariants $(w_{3D}^{LL}, w_{3D}^{UR})$ enabled by the sublattice symmetry, which obey the sum rule in Eq. (12). All invariants reverse sign for $\lambda < 0$. The star indicates the values $M = +3, \delta = +1$ considered throughout the manuscript, and the orange line indicates the range of parameter treated analytically for $k_x = k_y = 0$ in Supplementary Notes 2.B. and 2.C. **c** Color scheme for w_{3D} utilized in the previous panels.

where $h_{\text{block},i} \equiv \partial_{k_i} h_{\text{block}}$. Therefore, the total winding number can be expressed as a difference of winding numbers over the two blocks,

$$w_{3D} = w_{3D}^{LL} - w_{3D}^{UR}. \tag{12}$$

The existence of two integer point-gap invariants in the presence of sublattice symmetry is consistent with the classification tables, such as in Ref. 2. (Note that for non-Hermitian systems the definition of *sublattice symmetry* differs from the definition of *chiral symmetry*! We also remark that for Hermitian systems $w_{3D}^{LL} = w_{3D}^{UR}$, such that w_{3D} vanishes.)

In the following we indicate the the pair of winding numbers as $(w_{3D}^{LL}, w_{3D}^{UR})$, and we investigate the phase diagram in the parameter space of (M, λ, δ). Note that the two blocks can be expressed as

$$h_{LL}(\mathbf{k}; M, \lambda, \delta) = h_0(\mathbf{k}; M + \delta, \lambda) \qquad \text{and} \qquad h_{UR}(\mathbf{k}; M, \lambda, \delta) = h_0(\mathbf{k}; M - \delta, \lambda) \tag{13}$$

where

$$h_0(\mathbf{k}; m, \lambda) = \Big(\sum_{j=x,y,z} \cos k_j - m \Big) \sigma_0 + \mathrm{i}\lambda \sum_{j=x,y,z} \sin k_j \sigma_j, \tag{14}$$

such that both topological invariants can be extracted by studying the properties of a single block. The point gap of h_0 closes when for some $\mathbf{k}$ the determinant vanishes, which corresponds to a pair of conditions

$$\sum_{j=x,y,z} \cos k_j = m \qquad \text{and} \qquad \lambda^2 \Big(\sum_{j=x,y,z} \sin^2 k_j \Big) = 0. \tag{15}$$

Assuming $\lambda \neq 0$, the latter condition is fulfilled at TRIMs, in which case the first condition can only be satifies for $m \in \{\pm 1, \pm 3\}$. This observation fixes the boundaries of the phase diagram. Computing the winding number w_{3D}^0 then reveals the phase diagram displayed in Supplementary Fig. 1a. The phase diagram of the Hamiltonian in Eq. (9) is then obtained easily by substituting for the two blocks $m \mapsto M \pm \delta$, and is displayed in Supplementary Fig. 1b.

B. Slab Hamiltonian and the infernal point

In this section we first present the Hamiltonian for a slab geometry with open boundary condition in z-direction. We use the obtained Hamiltonian to reveal the macroscopic defectiveness at zero surface momentum, which we term

infernal point. Adopting the rotated basis of Eq. (9) the ETI slab Hamiltonian can be expressed in a block form as

$$H_{\rm slab}(k_x,k_y;M,\lambda,\delta;N)=\begin{pmatrix} H_0 & H_+ & \mathbb{0} & \mathbb{0} & \cdots & \mathbb{0} & \mathbb{0} & \mathbb{0} \\ H_- & H_0 & H_+ & \mathbb{0} & \cdots & \mathbb{0} & \mathbb{0} & \mathbb{0} \\ \mathbb{0} & H_- & H_0 & H_+ & \cdots & \mathbb{0} & \mathbb{0} & \mathbb{0} \\ \mathbb{0} & \mathbb{0} & H_- & H_0 & \ddots & \mathbb{0} & \mathbb{0} & \mathbb{0} \\ \vdots & \vdots & \vdots & \ddots & \ddots & \ddots & \vdots & \vdots \\ \mathbb{0} & \mathbb{0} & \mathbb{0} & \mathbb{0} & \ddots & H_0 & H_+ & \mathbb{0} \\ \mathbb{0} & \mathbb{0} & \mathbb{0} & \mathbb{0} & \cdots & H_- & H_0 & H_+ \\ \mathbb{0} & \mathbb{0} & \mathbb{0} & \mathbb{0} & \cdots & \mathbb{0} & H_- & H_0 \end{pmatrix} \tag{16}$$

where N is the number of layers (blocks), the diagonal blocks are

$$H_0(k_x,k_y;M,\lambda,\delta)=\begin{pmatrix} 0 & 0 & \tilde{f}(k_x,k_y;M)+\delta & -\mathrm{i}\lambda\sin k_x-\lambda\sin k_y \\ 0 & 0 & -\mathrm{i}\lambda\sin k_x+\lambda\sin k_y & \tilde{f}(k_x,k_y;M)+\delta \\ \tilde{f}(k_x,k_y;M)-\delta & \mathrm{i}\lambda\sin k_x+\lambda\sin k_y & 0 & 0 \\ \mathrm{i}\lambda\sin k_x-\lambda\sin k_y & \tilde{f}(k_x,k_y;M)-\delta & 0 & 0 \end{pmatrix} \tag{17}$$

where $\tilde{f}(k_x,k_y;M)=\cos k_x+\cos k_y-M$, and the next-to-diagonal blocks are

$$H_+=\begin{pmatrix} 0 & 0 & \frac{1-\lambda}{2} & 0 \\ 0 & 0 & 0 & \frac{1+\lambda}{2} \\ \frac{1+\lambda}{2} & 0 & 0 & 0 \\ 0 & \frac{1-\lambda}{2} & 0 & 0 \end{pmatrix} \quad\text{and}\quad H_-=\begin{pmatrix} 0 & 0 & \frac{1+\lambda}{2} & 0 \\ 0 & 0 & 0 & \frac{1-\lambda}{2} \\ \frac{1-\lambda}{2} & 0 & 0 & 0 \\ 0 & \frac{1+\lambda}{2} & 0 & 0 \end{pmatrix}=H_+^\top. \tag{18}$$

To proceed, we further consider $\lambda=+1$, and $M=2+\delta$, which includes the case $M=+3$ and $\delta=+1$ considered in the main text as a special case. (An analogous analytical treatment is also possible for $M=2-\delta$.) We keep the number of layers N as a free variable.

For the specified parameters, we aim to derive the spectrum for $k_x=k_y=0$ analytically by analyzing the characteristic polynomial $\det[H_{\rm slab}(k_x{=}0,\ k_y{=}0;\ M{=}2{+}\delta,\ \lambda{=}{+}1,\ \delta;\ N)-E\mathbb{1}]$. For these parameters, the diagonal block of $(H_{\rm slab}-E\mathbb{1})$ simplifies to

$$H_0-E\mathbb{1}=\begin{pmatrix} -E & 0 & 0 & 0 \\ 0 & -E & 0 & 0 \\ -2\delta & 0 & -E & 0 \\ 0 & -2\delta & 0 & -E \end{pmatrix} \quad\text{with inverse}\ \ [H_0-\lambda\mathbb{1}]^{-1}=\frac{1}{E^2}\begin{pmatrix} -E & 0 & 0 & 0 \\ 0 & -E & 0 & 0 \\ 2\delta & 0 & -E & 0 \\ 0 & 2\delta & 0 & -E \end{pmatrix}, \tag{19}$$

and the next-to-diagonal blocks reduce to

$$H_+=\begin{pmatrix} 0&0&0&0 \\ 0&0&0&1 \\ 1&0&0&0 \\ 0&0&0&0 \end{pmatrix} \quad\text{and}\quad H_-=\begin{pmatrix} 0&0&1&0 \\ 0&0&0&0 \\ 0&0&0&0 \\ 0&1&0&0 \end{pmatrix}=H_+^\top. \tag{20}$$

For brevity, we will write $H_{\rm slab}(0,0,;2+\delta,1,\delta;N)-E\mathbb{1}\equiv\tilde{H}_{\rm slab}(N)$. Motivated by Ref. 3, we use Schur's determinant identity

$$\det\begin{pmatrix} A & B \\ C & D \end{pmatrix}=\det(D)\det(A-B\cdot D^{-1}\cdot C) \tag{21}$$

where A and D are square matrices (not necessarily of the same dimension), and it is assumed that $\det D\neq 0$. Setting $D=H_0$ corresponding to the bottom-right 4×4 block of $\tilde{H}_{\rm slab}(N)$, we find

$$-B\cdot D^{-1}\cdot C=\begin{pmatrix} \ddots & \vdots & \vdots \\ \cdots & \mathbb{0} & \mathbb{0} \\ \cdots & \mathbb{0} & M \end{pmatrix} \quad\text{with}\ \ M=-H_+\cdot[H_0^{-1}-E\mathbb{1}]^{-1}\cdot H_-=\begin{pmatrix} 0&0&0&0 \\ 0&E^{-1}&0&0 \\ 0&0&E^{-1}&0 \\ 0&0&0&0 \end{pmatrix} \tag{22}$$

and that

$$\det \tilde{H}_{\mathrm{slab}}(N) = E^4 \det[\tilde{H}_{\mathrm{slab}}(N-1) - B \cdot D^{-1} \cdot C]. \tag{23}$$

The computation unexpectedly simplifies at the next step of applying the Shur's determinant identity. Note that the bottom-right block of $(\tilde{H}_{\mathrm{slab}}(N-1) - B \cdot D^{-1} \cdot C)$ is

$$D' = \begin{pmatrix} -E & 0 & 0 & 0 \\ 0 & E^{-1} - E & 0 & 0 \\ -2\delta & 0 & E^{-1} - E & 0 \\ 0 & -2\delta & 0 & -E \end{pmatrix} \quad \text{with} \;\; \det D' = (E^2 - 1)^2, \tag{24}$$

and with the same product $-B' \cdot (D')^{-1} \cdot C'$ as obtained in the previous step in Eq. (22). We therefore obtain

$$\det \tilde{H}_{\mathrm{slab}}(j+2) = (E^2-1)^2 \det[\tilde{H}_{\mathrm{slab}}(j+1) - B^{(j)} \cdot (D')^{-1} \cdot C^{(j)}] \tag{25}$$

where

$$C^{(j)} = \big(\underbrace{\mathbb{0}\ \mathbb{0}\ \cdots\ \mathbb{0}}_{j \text{ blocks}}\, H_-\big) \qquad \text{and} \qquad B^{(j)} = \left(C^{(j)}\right)^\top, \tag{26}$$

which remains valid for all $N - 3 \geq j \geq 0$. In the last step, knowing that $\tilde{H}_{\mathrm{slab}}(1) = H_0 - E\mathbb{1}$, we find that $(H_{\mathrm{slab}}(1) - H_+ \cdot (D')^{-1} H_-) = D'$ with the determinant shown in Eq. (24). Altogether, we obtain

$$\det \tilde{H}_{\mathrm{slab}}(N) = E^4 (E^2-1)^{2(N-1)}. \tag{27}$$

Therefore, for the chosen parameters ($k_x = k_y = 0$, $\lambda = 1$, and $M - \delta = 2$), the characteristic polynomial has $2N - 2$ roots at $E = +1$, $2N - 2$ roots at $E = -1$, and 4 roots at $E = 0$.

Knowing the eigenenergies, it is also possible to search for the corresponding eigenstates. Parametrizing a generical eigenvector as

$$\psi = \left(\ldots,\ d^{(j-1)}\ \mid\ a^{(j)},\ b^{(j)},\ c^{(j)},\ d^{(j)}\ \mid\ a^{(j+1)}\ \ \ldots\right)^\top \qquad \text{where} \quad 1 \leq j \leq N \tag{28}$$

the eigentates correspond to solutions of

$$\begin{aligned} Ea^{(1)} &= 0 \\ Eb^{(N)} &= 0 \\ Ec^{(N)} &= -2\delta a^{(N)} \\ Ed^{(1)} &= -2\delta b^{(1)} \end{aligned} \qquad \text{and} \qquad \begin{aligned} Ea^{(j)} &= c^{(j-1)} && \text{for } 2 \leq j \leq N \\ Eb^{(j)} &= d^{(j+1)} && \text{for } 1 \leq j \leq N-1 \\ Ec^{(j)} &= -2\delta a^{(j)} + a^{(j+1)} && \text{for } 1 \leq j \leq N-1 \\ Ed^{(j)} &= b^{(j-1)} - 2\delta b^{(j)} && \text{for } 2 \leq j \leq N. \end{aligned} \tag{29}$$

Assuming $\delta \neq 0$, such that the Hamiltonian is non-Hermitian, there are (not caring about the normalization) the following linearly independent eigenstates:

- two eigenstates at $E = 0$, localized on opposite surfaces, namely $(0,0,0,1|0,\ldots)^\top$ and $(\ldots 0|0,0,1,0)^\top$ (both have a single non-vanishing component).
- a pair of eigenstates at each $E = \pm 1$, also localized on opposite surfaces, namely $(0,\pm 1, 0, -2\delta|0,0,0,1|0\ldots)^\top$ and $(\ldots 0|0,0,1,0|\pm 1, 0, -2\delta, 0)^\top$ (they all have exactly three non-vanishing components).

Altogether, the Hamiltonian with dimension $4N$ has only 6 distinct eigenstates, indicating a huge defectiveness. (We remark that for $\delta = 0$ the Hamiltonian becomes Hermitian, and contains $4N$ distinct eigenstates as expected.)

C. Expansion near the infernal point

We now extend to a small but finite k_x and k_y by assuming a linear expansion of $\sin k_i \approx k_i$ and $\cos k_i \approx 1$ in Eq. (17), while keeping $\lambda = 1$ and $M = \delta + 2$. This changes the diagonal block (with substracted $\lambda \mathbb{1}$) to

$$H_0 - E\mathbb{1} = \begin{pmatrix} -E & 0 & 0 & -\mathrm{i}k_x - k_y \\ 0 & -E & -\mathrm{i}k_x + k_y & 0 \\ -2\delta & \mathrm{i}k_x + k_y & -E & 0 \\ \mathrm{i}k_x - k_y & -2\delta & 0 & -E \end{pmatrix}. \tag{30}$$

At this stage an exact analytic solution becomes infeasible. Nevertheless, one can still deduce some properties about the dispersion near the zero-energy states of the infernal point by considering the expansion of the characteristic polynomial for small values of k_x, k_y and E. We only need to keep the contributions to the polynomial that are of

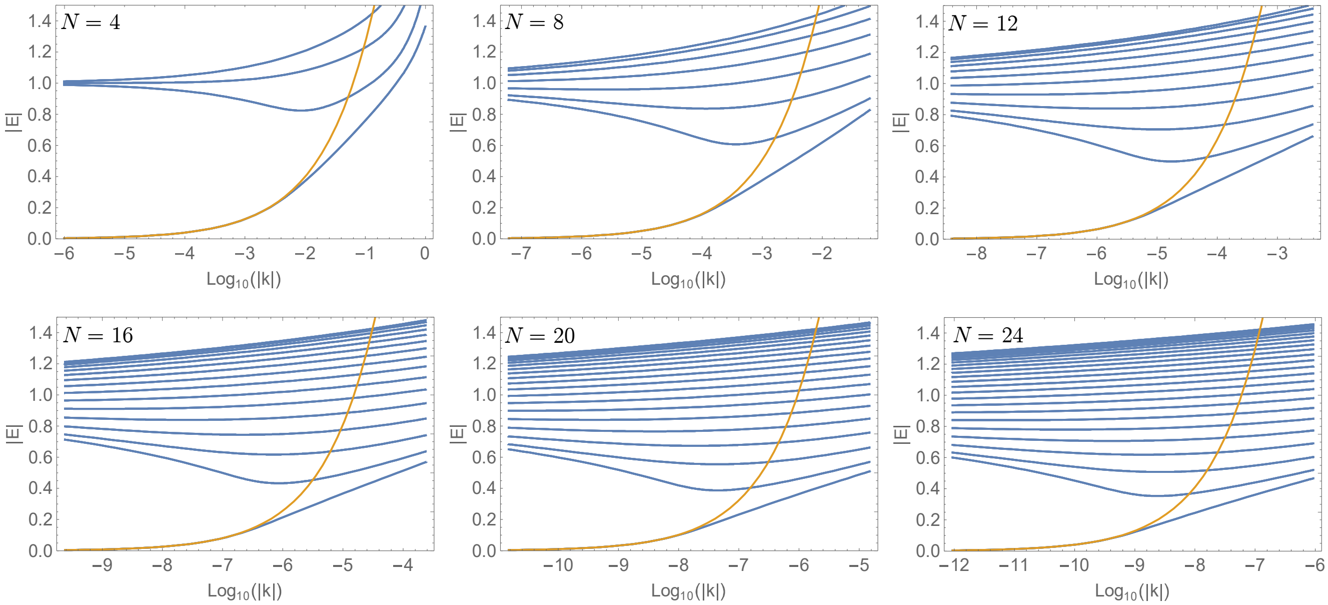

Supplementary Figure 2. **Dispersion of the states near the infernal point $k_x = k_y = 0$, $M = 3$, $\delta = \lambda = 1$ for several small values of N.** The blue lines correspond to numerically found eigenvalues, while the yellow line indicates the theoretically predicted dispersion of the zero-energy states in Eq. (34). We observe that the theoretical result approximates the spectrum well for energies $E \lesssim 0.2$, which corresponds to an exponentially shrinking interval of momenta around the infernal point (notice the logarithmic scale on the horizontal axis). In the thermodynamic limit we expect the spectrum of the slab Hamiltonian with open boundary condition to exhibit a discontinuous jump between $E = 0$ and a finite value of energy.

the lowest order in these variables. These correspond to the following two contributions to the determinant,

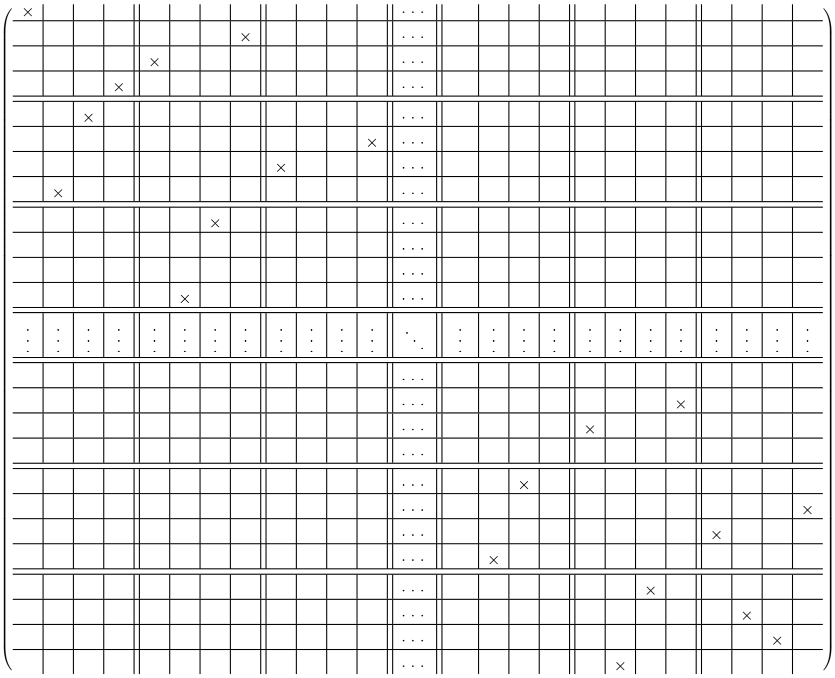

$$= +E^4 \tag{31}$$

and

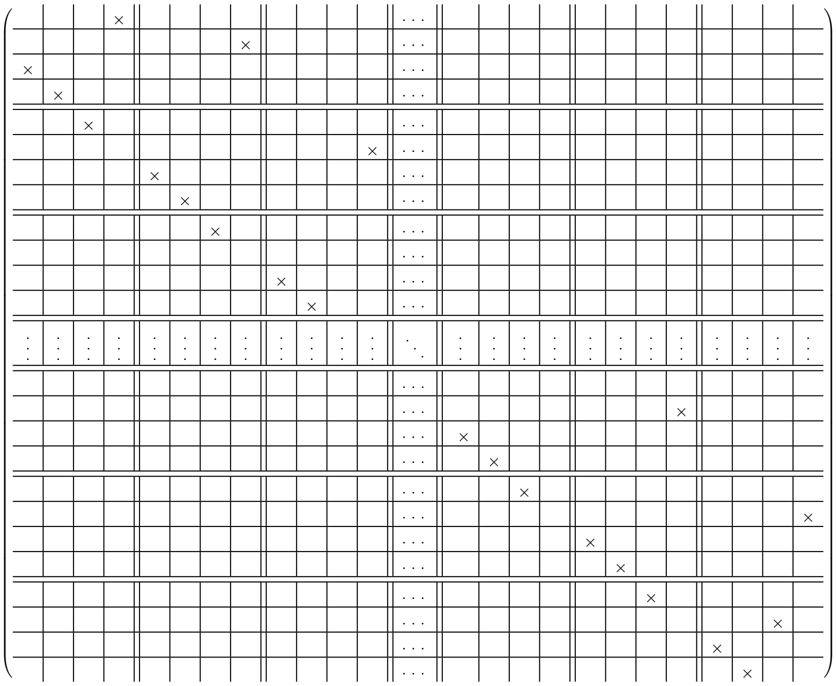

$$= (2\delta)^{2N}(k_x^2 + k_y^2). \tag{32}$$

As all the other terms are of higher order in either E or $(k_x^2+k_y^2)$ than the two listed terms, there exists a neighborhood around $k_x = k_y = 0$ where these two terms are dominant. In this neighborhood, the dispersion of the previously identified zero-energy states is obtained by solving for $\{E\}_{i=1}^{4}$ in the equation

$$(E - E_1)(E - E_2)(E - E_3)(E - E_4) = E^4 + (2\delta)^{2N}(k_x^2 + k_y^2). \tag{33}$$

The solution is

$$E_{1,2,3,4} = \pm\sqrt{\pm \mathrm{i}}(2\delta)^{N/2}\sqrt{k} \qquad \text{where} \quad k = \sqrt{k_x^2 + k_y^2}. \tag{34}$$

Note that the coefficient in the derived square-root dispersion grows exponentially with the number of layers, and becomes *infinitely steep* in the thermodynamic limit $N \to \infty$.

SUPPLEMENTARY NOTE 3: HIGHER-ORDER EXCEPTIONAL POINT ON THE SURFACE OF ETI

We showed in the main text that by tuning the g-factor angle α of the model in Eq. (1) in the range $[0, \pi/2]$, the surface states filling the point-gap region continuously evolve from a single-sheet covering (for $\alpha = \pi/2$) to a pair of bands connected with an exceptional point (EP) at $E = 0$ (for $\alpha = 0$) (cf. Fig. 2a and b). Rather than encountering some sharp transition, this is a simple consequence of the EP moving/leaving the point-gap region from/to the bulk energy bands. In this section, we show that the evolution of the surface states with α is, in fact, *even richer*, as it exhibits a collision and bouncing of two elementary (so-called *second-order*) EPs, with the critical point corresponding to a *third-order* EP[4]. For brevity, we refer to these two types of band degeneracy as EP2, resp. EP3. Recall that an nth order exceptional point (EPn) is a singularity where n bands become defective, and disperse as $(k_x + ik_y)^{1/n}$.

To reveal the presence of the EPs, we plot in Supplementary Fig. 4 the surface states across the critical point in a way similar to Fig 2 a) and b) of the main text. More specifically, we consider a slab geometry with $N = 20$ layers, and we take a regular square grid of momentum points with $\Delta k_x = \Delta k_y = 2\pi/800$ (we find the region $|k_{x,y}| < 2\pi/10$ to be sufficiently large to include all the surface states filling the point-gap region) inside the two-dimensional Brillouin zone. We indicate all the identified eigenstates as points inside the complex energy plane, and color them blue to red according to their localization (following their inverse participation ratio $\text{IPR}[|\psi\rangle] = \sum_i |\psi(i)|^4 / \sum_i |\psi(i)|^2$ where $\psi(i)$ are components of the right eigenstate $|\psi\rangle$).

Crucially, we also consider Cartesian lines in **k**-space by including two additional points along each bond of the square lattice, following the schematics of Supplementary Fig. 3a. The image of these Cartesian lines is visible in the surface-state energies of Fig. 2a and b and of Supplementary Fig. 4 as a deformed square grid. From the deformation of the square grid one can deduce how a piece of the surface Brillouin zone is pasted onto the complex energy plane. Besides continuous deformations, one observes that the square grid occasionally exhibits disclination defects. We argue that these indicate various types of exceptional points on the surface of ETI at the corresponding complex energy.

Let us first consider an isolated n^{th} order exceptional point (EPn) occurring inside one of the squares of the momentum grid [Supplementary Fig. 3(b, orange)]. Then the bands form a Riemann sheet that wind n times around the central singularity until it connects back onto itself. Therefore, one needs to go n times along the edges of the square (i.e. make $4n$ right turns along the orange path γ_0) before returning to the original state. This is revealed as an octagon (i.e. 2π disclination) for EP2, and as a dodecagon (i.e. 4π disclination) for EP3.

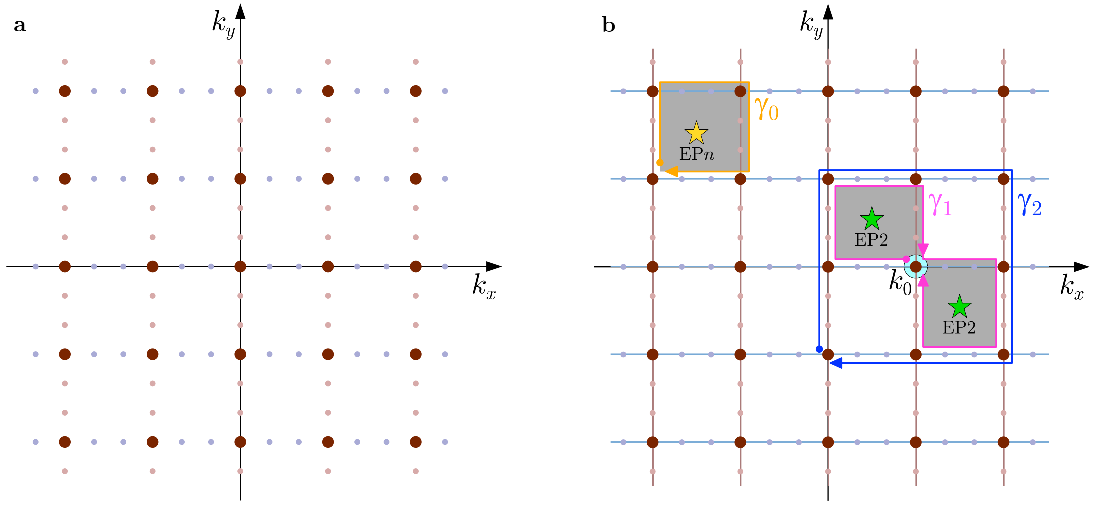

Supplementary Figure 3. **Momentum grid and paths for the surface spectra in this work.** **a** The grid of points in momentum space for which we plot the surface spectra in Fig. 2a and b of the main text and in Supplementary Fig. 4 below. (**b**, orange) Assuming the presence of an nth order exceptional point (EPn, yellow star), one needs to circumvent it n times until the energy band connects back onto itself. As a consequence, the surface-states spectrum along the square path γ_0 looks like a polygon with $4n$ sides in the complex energy plane. (**b**, magenta) If there are two nearby second-order exceptional points (EP2s, green stars), one could enclose each one of them in sequence by following the magenta path γ_1. The energy spectrum along γ_1 reveals whether the two EPs are formed by *the same* pair of bands, or by two *different* pairs of adjacent bands. (**b**, blue) If three bands participate in the formation of the two adjacent EP2s, the spectrum along γ_2 is equivalent to the spectrum along γ_0 for $n = 3$. Therefore, the merging of two EP2s results in an EP3. For a detailed discussion see Supplementary Note 3.

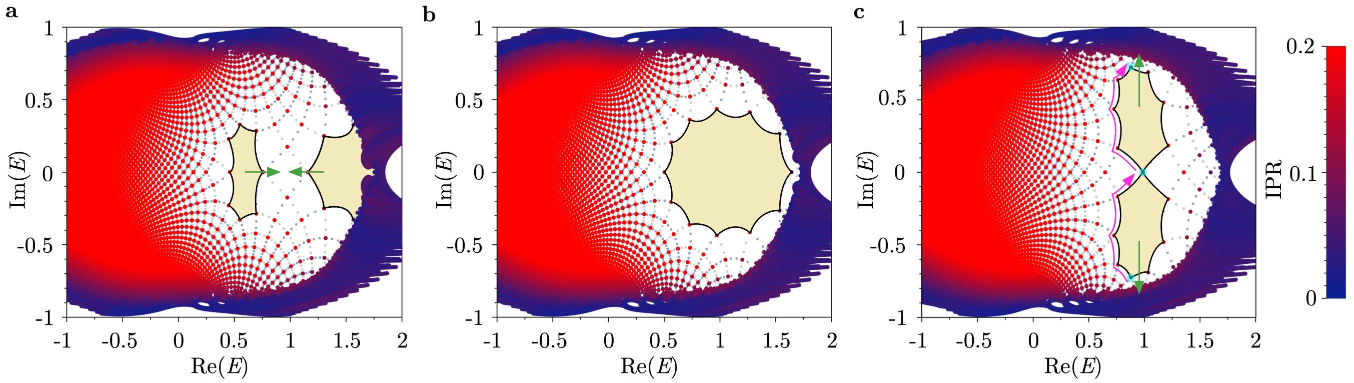

Supplementary Figure 4. **Surface states for (a-c) g-factor angles $\alpha = 0.57$, 0.64 and 0.72.** We indicate surface states at momentum resolution $\delta k = 2\pi/800$ in both $k_{x,y}$-directions in red-to-blue tones according to their inverse participation ratio (IPR). The smaller light-gray points with a finer resolution $\delta k = 2\pi/2400$ represent the momentum mesh; more specifically, they indicate the band energies along lines parallel with either k_x or k_y axis (cf. Supplementary Fig. 3a). The presence of 'ordinary' (second-order) exceptional points (EP2s) is revealed by an octagon shape with eight right angles [light yellow in a and c], and their motion with increasing α is indicated by the green arrows. Two EPs collide and bounce at $\alpha \approx 0.64$, while forming a third-order exceptional point (EP3) at the collision, revealed by a dodecagon shape with twelve right angles [light yellow in b]. The meaning of the magenta arrows, of the cyan dots in panel c, and the detailed reasoning behind the occurrence of the polygons are clarified in the text of Supplementary Note 3.

Since both an octagon and a dodecagon are observed in the panels of Supplementary Fig. 4, one interprets the evolution of the surface states with increasing α as follows. First (Fig. 2b, $\alpha = 0$) there is a single EP2 at $E = 0$. With increasing α the EP2 moves to higher energies, while at the same time (Supplementary Fig. 4a, $\alpha = 0.57$) another EP2 enters the point-gap region from the bulk states at $E \approx 1.6$. The two EP2s collide (Supplementary Fig. 4b, $\alpha = 0.64$) at $E \approx 1.1$, apparently forming an EP3 at the critical point. The EP2s then bounce and depart symmetrically from the real energy axis (Supplementary Fig. 4c, $\alpha = 0.72$), until they both leave the point-gap region, leaving behind only a single-sheet covering surface state (Fig. 2a, $\alpha = \pi/2$). [We remark that without additional analysis, our plots cannot be directly used to tell the momentum (k_x, k_y) at which the EPs are formed.]

To analyze the situation near the critical point (Supplementary Fig. 4b, $\alpha = 0.64$) in more detail, we consider a pair of EP2s in two nearby infinitesimal squares of the momentum grid [Supplementary Fig. 3(b, magenta)]. We construct the magenta path γ_1 that starts at $\mathbf{k}_0$ (cyan dot) between the EP2s, then (i) encloses the first EP2, then (ii) returns to $\mathbf{k}_0$, then (iii) encloses the second EP2, until finally (iv) it returns to $\mathbf{k}_0$ again. We argue that the spectrum along γ_1 signals whether the two EPs are formed by the same pair of energy bands, or by three contiguous energy bands. To understand this, note that circumventing an EP2 results in a permutation of two band indices, symbolically $(\overline{1,2}) \mapsto (2,1)$. If the two EP2s were formed by the same pair of bands, then the two permutations along γ_1 act, in sequence, as $(\overline{1,2}) \mapsto (\overline{2,1}) \mapsto (1,2)$ (the overline indicates the two bands which are permuted at the next step), meaning that the total permutation is trivial, and that the initial and the final state encountered along γ_1 at $\mathbf{k}_0$ are the same.

We apply this reasoning to the pair of EP2s visible in Supplementary Fig. 4c after the collision. Starting at the cyan point on the bottom, and departing in the indicated direction, we follow the magenta path in Supplementary Fig. 3b (performing in sequence: three right turns, one left turn, and three right turns). The three cyan points in Supplementary Fig. 4c correspond to the same momentum $\mathbf{k}_0$, but lie at three distinct energies. Manifestly, the initial and the final state are different. This implies that there are *three surface bands* at play, and that the two EP2s are formed by different pairs of bands.

We finally explain why the merging of two EP2s results in an EP3. For that, note that the magenta path γ_1 is continuously deformable into the square blue path γ_2 [Supplementary Fig. 3(b, blue)]. The total permutation of bands along the paths γ_1 and γ_2 is the same, namely $(\overline{1,2},3) \mapsto (2,\overline{1,3}) \mapsto (2,3,1)$. Note that this is a cyclic permutation, exactly as expected for an EP3. Therefore, the spectrum along the square path γ_2 that encircles two EP2 formed by three adjacent bands looks the same as expected for the square path γ_0 encircling an EP3. This correspondence suggests the formation of an exact EP3 at the critical point[4].

SUPPLEMENTARY NOTE 4: BULK INVARIANT

In this section, we relate the bulk invariant w_{3D} of a 3D non-Hermitian Hamiltonian with a point gap [Eq. (5) of the main text] to the flow of Chern numbers around the point gap [Eq. (2) of the main text]. Note that the same result has been obtained in Ref. 5. Our argument is divided into four parts, distributed over the four subsection below. First, in Supplementary Note 4.A. we deform the non-Hermitian Hamiltonian into a Floquet unitary form by employing the polar decomposition while preserving the topological invariants. Second, in Supplementary Note 4.B., we depart from the unitary form and review the proof from Ref. 6 which derives the equivalence of Eqs. (5) and (2) for the special case of two-band models with vanishing w_{1D}. Finally, by appealing to the topological nature of the integrals in Eqs. (5) and (2) we show that the correspondence generalizes to models with arbitrarily many bands [Supplementary Note 4.C.] and with arbitrary values of w_{1D} along the three directions of the BZ torus [Supplementary Note 4.D.].

A. Mapping the non-Hermitian Hamiltonian to a Floquet operator via polar decomposition

We first review the arguments presented in Appendix G of Ref. 1, which relate a non-Hermitian Hamiltonian with a point-gap to a unitary Floquet operator. To do so, recall that a non-singular matrix $H(\mathbf{k}) - E\mathbb{1}$ (i.e. with a point gap at energy $E \in \mathbb{C}$) has a polar decomposition. Setting for simplicity $E = 0$, this corresponds to

$$H(\mathbf{k}) = U(\mathbf{k})P(\mathbf{k}), \tag{35}$$

where $P(\mathbf{k}) = [H(\mathbf{k})^\dagger H(\mathbf{k})]^{1/2}$ is a positive-definite self-adjoint matrix, and $U(\mathbf{k}) = H(\mathbf{k})P(\mathbf{k})^{-1}$ is a unitary matrix. The decomposition into $U(\mathbf{k})$ and $P(\mathbf{k})$ is unique and depends continuously on $\mathbf{k}$ when $H(\mathbf{k})$ is continuous (which is the case for any physical system).

Importantly, if A and B are positive-definite self-adjoint matrices, then the linear combination $tA + (1-t)B$ for $t \in [0, 1]$ is also a positive-definite matrix. Setting these matrices specifically to $A(\mathbf{k}) = \mathbb{1}$ and $B = P(\mathbf{k})$, we find that

$$F(\mathbf{k}, t) = U(\mathbf{k})\,[t\mathbb{1} + (1-t)P(\mathbf{k})] \tag{36}$$

is a *homotopy* that continuously deforms $F(\mathbf{k}, 0) = H(\mathbf{k})$ to $F(\mathbf{k}, 1) = U(\mathbf{k})$ while preserving the point-gap at all $\mathbf{k}$ and t [i.e. $\det F(\mathbf{k}, t) \neq 0$]. Furthermore, since we did not assume any symmetry of $H(\mathbf{k})$, we are not required to check for symmetries of $U(\mathbf{k})$. As a consequence, $H(\mathbf{k})$ and $U(\mathbf{k})$ share all point-gap invariants.

We conclude that for each non-Hermitian $H(\mathbf{k})$ with a point gap there is a canonically associated unitary $U(\mathbf{k})$. The continuous deformation that evolves the first into the latter changes the eigenstates and normalizes eigenvalues to absolute value 1, but it preserves all point-gap invariants. Besides relating non-Hermitian and Floquet topology, the obtained equivalence is particularly useful for analytical manipulations. Note that a unitary matrix is never defective, meaning that it always has a complete set of eigenstates. In particular, the generic band singularity of $H(\mathbf{k})$ is an exceptional line, while that of $U(\mathbf{k})$ is a Weyl point. This implies that $U(\mathbf{k})$ has the usual spectral decomposition at each $\mathbf{k}$, which is impossible for non-Hermitian Hamiltonians at exceptional degeneracies.

B. Relating w_{3D} to Chern numbers in two-band models with $w_{1D} = 0$

For a Floquet unitary $U(\mathbf{k})$, the eigenvalues become $\mathsf{U}(1)$ phases, i.e. $\lambda^a_{\mathbf{k}} = e^{i\varepsilon^a_{\mathbf{k}}}$. We call below the complex argument $\varepsilon^a_{\mathbf{k}}$ the *quasi-energy* of ath band, and we refer to the (counter-clockwise) direction along the unitary circle as the (positive) *quasi-energy direction*. It is possible to consider all the $\mathbf{k}$-points with fixed quasi-energy,

$$\mathrm{FS}(\mu) = \{\mathbf{k} \in \mathrm{BZ}\,|\,\exists a : \varepsilon^a_{\mathbf{k}} = \mu \pmod{2\pi}\}, \tag{37}$$

which corresponds to a Fermi surface of $U(\mathbf{k})$ at quasi-energy μ. The orientation of the Fermi surface $\mathrm{FS}(\mu)$ is defined to be along the increasing quasi-energy. In the remainder of the present subsection we consider two-band models, i.e., $U(\mathbf{k}) : \mathrm{BZ} \to \mathsf{U}(2)$. Furthermore, we assume that w_{1D} is trivial for all directions along the Brillouin zone torus, in which case the Floquet unitary can be continuously deformed into $\mathsf{SU}(2)$. For cases when these two conditions are fulfilled, it was shown by Ref. 6 that

$$w_{3D} = C_{\mathrm{FS}(\mu)}, \tag{38}$$

i.e. the winding number of $U(\mathbf{k})$ is equal to the Chern number on the Fermi surface $\mathrm{FS}(\mu)$ for an arbitrary choice of Fermi quasi-energy μ (i.e., equivalently, to the flow of Berry curvature in the quasi-energy direction around the point gap). Below, we review the arguments of Ref. 6 that lead to the validity of Eq. (38) in two-band models. These arguments are generalized to the case of many bands and of non-vanishing $w_{1\mathrm{D}}$ in the subsequent Supplementary Notes 4.C. and 4.D.

Starting with a generic two-band unitary, note that $\mathsf{U}(2) = \mathsf{U}(1) \ltimes \mathsf{SU}(2)$ (semi-direct product). The decomposition of the Floquet unitary $U(\mathbf{k})$ into the two components is not unique: while the $\mathsf{U}(1)$-component of $U(\mathbf{k})$ is simply $\det[U(\mathbf{k})]$, the $\mathsf{SU}(2)$-component $\tilde{U}(\mathbf{k}) = U(\mathbf{k})/\sqrt{\det[U(\mathbf{k})]}$ has a $\pm$ sign ambiguity. Therefore, we choose to first prove Eq. (38) for the class of unitaries $U(\mathbf{k})$ that do not wind non-trivially along the $\mathsf{U}(1)$ component (i.e., for which $w_{1\mathrm{D}}$ is trivial for all directions around the Brillouin-zone torus). In such cases it is possible to choose the branch of the square root globally, implying that $\tilde{U}(\mathbf{k})$ is a global and smooth function on the entire Brillouin zone. Furthermore, the assumption also allows us to continuously deform the $\mathsf{U}(1)$-component to a constant map [e.g., to $1 \in \mathsf{U}(1)$], in which case the Floquet unitary operator $U(\mathbf{k})$ is continuously deformed to *special* unitary $\tilde{U}(\mathbf{k})$. Because of the continuity, such a deformation preserves both the left- and the right-hand side of Eq. (38).

The general $\mathsf{SU}(2)$ matrix can be decomposed using the identity matrix and the Pauli matrices as

$$\tilde{U}(\mathbf{k}) = n_0(\mathbf{k})\mathbb{1} - \mathrm{i}\,[n_x(\mathbf{k})\sigma_x + n_y(\mathbf{k})\sigma_y + n_z(\mathbf{k})\sigma_z] \tag{39}$$

with norm

$$||n||^2 = n_0^2 + n_x^2 + n_y^2 + n_z^2 = 1 \tag{40}$$

at each $\mathbf{k}$, indicating the well-known property that as a topological space $\mathsf{SU}(2) \cong S^3$. We use this simple expression for a general $\mathsf{SU}(2)$ matrix to simplify the formula for $w_{3\mathrm{D}}$.

Noting that $\tilde{U}^\dagger(\mathbf{k})$ is obtained from $\tilde{U}(\mathbf{k})$ simply by flipping the sign $-\mathrm{i} \mapsto +\mathrm{i}$, one easily checks that

$$\begin{aligned}\tilde{U}^{-1}\partial_k\tilde{U} = \left(\Sigma_{a\in\{0,x,y,z\}} n_a\partial_k n_a\right)\mathbb{1} &+ \mathrm{i}\,[(\partial_k n_0)n_x - (\partial_k n_x)n_0 - (\partial_k n_y)n_z + (\partial_k n_z)n_y]\,\sigma_x \\ &+ \mathrm{i}\,[(\partial_k n_0)n_y - (\partial_k n_y)n_0 - (\partial_k n_z)n_x + (\partial_k n_x)n_z]\,\sigma_y \\ &+ \mathrm{i}\,[(\partial_k n_0)n_z - (\partial_k n_z)n_0 - (\partial_k n_x)n_y + (\partial_k n_y)n_x]\,\sigma_z,\end{aligned} \tag{41}$$

where we dropped the $\mathbf{k}$-arguments for brevity. Note that the obtained prefactor of $\mathbb{1}$ is simply $\frac{1}{2}\partial_k||n(\mathbf{k})||^2 = 0$ according to Eq. (40), and the term drops out. We thus rewrite

$$\tilde{U}^{-1}\partial_k\tilde{U} = \sum_{a\in\{x,y,z\}} \mathrm{i}\,[(\partial_k n_0)n_a - (\partial_k n_a)n_0 - \epsilon_{abc}(\partial_k n_b)n_c]\sigma_a. \tag{42}$$

Furthermore, since $\tilde{U}^{-1}\tilde{U} = \mathbb{1}$, we can rewrite $\tilde{U}^{-1}\partial_i U = -(\partial_i\tilde{U}^{-1})\tilde{U}$ for one of the derivatives in Eq. (5) of the main text, such that the formula for the 3D winding number reduces to

$$\begin{aligned} w_{3\mathrm{D}} &= +\frac{1}{24\pi^2}\int_{\mathrm{BZ}} d^3\mathbf{k}\,\epsilon_{ijk}\mathrm{Tr}[(\partial_i\tilde{U}^{-1})\tilde{U}\tilde{U}^{-1}(\partial_j\tilde{U})\tilde{U}^{-1}(\partial_k\tilde{U})] && (43)\\ &= +\frac{1}{24\pi^2}\int_{\mathrm{BZ}} d^3\mathbf{k}\,\epsilon_{ijk}\mathrm{Tr}[(\partial_i\tilde{U}^{-1})(\partial_j\tilde{U})\tilde{U}^{-1}(\partial_k\tilde{U})] && (44)\end{aligned}$$

with implicit summation over $i,j,k \in \{k_x,k_y,k_z\}$. We further use that

$$(\partial_i\tilde{U}^{-1})(\partial_j\tilde{U}) = \sum_{a\in\{0,x,y,z\}}(\partial_i n_a)(\partial_i n_j)\mathbb{1} + \sum_{a\in\{x,y,z\}}(-\mathrm{i})\,[(\partial_i n_0)(\partial_j n_a) - (\partial_i n_a)(\partial_j n_0) - \epsilon_{ade}(\partial_i n_d)(\partial_j n_e)]\,\sigma_a. \tag{45}$$

We now substitute expressions (42) and (45) into Eq. (44). Since Pauli matrices have zero trace, the only combinations of terms that contribute to Eq. (44) are those that combine two copies of the same Pauli matrix (i.e., $\mathrm{Tr}[\sigma_a\cdot\sigma_a] = 2$). Therefore, the expression behind the integral of Eq. (44) equals

$$2\epsilon_{ijk}[(\partial_i n_0)(\partial_j n_a) - (\partial_i n_a)(\partial_j n_0) - \epsilon_{ade}(\partial_i n_d)(\partial_j n_e)][(\partial_k n_0)n_a - (\partial_k n_a)n_0 - \epsilon_{abc}(\partial_k n_b)n_c], \tag{46}$$

where i,j,k is summed over $\{k_x,k_y,k_z\}$, and a,b,c over $\{x,y,z\}$.

We would now like to expand the product of the square brackets in Eq. (46). One readily observes that many of the resulting terms vanish for symmetry reasons. On the one hand, combining any two terms without the $\epsilon_{a\ldots}$ Levi-Civita symbol results in expressions containing either $\epsilon_{ijk}(\partial_i n_0)(\partial_k n_0) = 0$ or $\epsilon_{ijk}(\partial_i n_a)(\partial_k n_a) = 0$ (or equivalent).

The cancellation arises after summation over the i, k indices due to taking the product of objects symmetric [i.e., $(\partial_i n_a)(\partial_k n_a)$] and antisymmetric (i.e., ϵ_{ijk}) in $i \leftrightarrow k$. Similarly, the combination involving $\epsilon_{abc}\epsilon_{ade} = (\delta_{bd}\delta_{ce} - \delta_{be}\delta_{ce})$ is also easily shown to combine the antisymmetric tensor ϵ_{ijk} with a combination of derivatives symmetric in $i \leftrightarrow k$ or $j \leftrightarrow k$, and therefore also vanishes. In summary, only the products that contain *exactly one* of the $\epsilon_{a\ldots}$ symbols are non-vanishing after summation, which (after also renaming $d \mapsto b$ and $e \mapsto c$) results in

$$2\epsilon_{ijk}\epsilon_{abc}[-(\partial_i n_0)(\partial_j n_a)(\partial_k n_b)n_c + (\partial_i n_a)(\partial_j n_0)(\partial_k n_b)n_c - (\partial_i n_b)(\partial_j n_c)(\partial_k n_0)n_a + (\partial_i n_b)(\partial_j n_c)(\partial_k n_a)n_0]. \tag{47}$$

After slight reordering of the terms and permuting the indices, the same expression is equivalently written as

$$2\epsilon_{ijk}\epsilon_{abc}[n_0(\partial_i n_a)(\partial_j n_b)(\partial_j n_c) - n_a(\partial_i n_0)(\partial_j n_b)(\partial_k n_c) + n_a(\partial_i n_b)(\partial_j n_0)(\partial_k n_c) - n_a(\partial_i n_b)(\partial_j n_c)(\partial_k n_0)] \tag{48}$$

which one should recognize as

$$\begin{aligned} &2\epsilon_{ijk}[\epsilon_{0bcd}n_0(\partial_i n_b)(\partial_j n_c)(\partial_j n_d) + \epsilon_{a0cd}n_a(\partial_i n_0)(\partial_j n_c)(\partial_k n_d) \\ &+ \epsilon_{ab0d}n_a(\partial_i n_b)(\partial_j n_0)(\partial_k n_d) + \epsilon_{abc0}n_a(\partial_i n_b)(\partial_j n_c)(\partial_k n_0)] \\ =\ &2\epsilon_{ijk}\epsilon_{abcd}n_a(\partial_i n_b)(\partial_j n_c)(\partial_k n_d). \end{aligned} \tag{49}$$

where a, b, c, d now range over the enlarged set $\{0, x, y, z\}$. Therefore, one obtains

$$w_{3\mathrm{D}} = \frac{1}{12\pi^2}\int_{\mathrm{BZ}} d^3\mathbf{k}\epsilon_{ijk}\epsilon_{abcd}n_a(\partial_i n_b)(\partial_j n_c)(\partial_k n_d) = \frac{1}{2\pi^2}\int_{\mathrm{BZ}} d^3\mathbf{k}\epsilon_{abcd}n_a(\partial_{k_x} n_b)(\partial_{k_y} n_c)(\partial_{k_z} n_d), \tag{50}$$

which is recognized as the wrapping number of the Brillouin zone torus around the 3-sphere. (Note that the normalization is also correct because $2\pi^2$ is exactly the surface area of the unit 3-sphere.)

The integrand in Eq. (50) is interpreted as the oriented area of the unit 3-sphere swiped by $\mathbf{n}(\mathbf{k}) = (n_0, n_x, n_y, n_z)$ as $\mathbf{k}$ is varied over the infinitesimal volume $d^3\mathbf{k}$. The orientation is important, namely it means that the area is being "covered" resp. "uncovered" depending on the sign of the integrand. A generic point $x_0 \in S^3$ is covered (uncovered) by the map $\tilde{U}(\mathbf{k}) : \mathrm{BZ} \mapsto S^3$ a number of times which we denote $\nu_+(x_0)$ $[\nu_-(x_0)]$. The geometric nature of the wrapping number (50) implies that

$$w_{3\mathrm{D}} = \nu_+(x_0) - \nu_-(x_0), \tag{51}$$

i.e., each $x_0 \in S^3$ is wrapped the same number of times if the orientation is taken into account. In a more technical language[7], the equal dimensionality of $\mathsf{SU}(2) \cong S^3$ and of $\mathrm{BZ} = T^3$ implies that the Pontryagin manifold of $\tilde{U} : \mathrm{BZ} \to \mathsf{SU}(2)$ [i.e. the framed pre-image $\tilde{U}^{-1}(x_0)$ of any point $x_0 \in \mathsf{SU}(2)$, with framing defined by pulling back a basis of tangent space $T_{x_0}\mathsf{SU}(2)$] is generically a set of discrete points $\{\mathbf{k}_i\}_{i\in\mathcal{I}}$ that depends on $\tilde{U}$ and x_0, each point carrying positive or negative orientation $\mathfrak{o}(\mathbf{k}_i)$ [the handedness of the pulled-back frame]. It follows from the Pontryagin-Thom construction that the index of the map $\tilde{U}$ is

$$w_{3\mathrm{D}} = \sum_i \mathfrak{o}(\mathbf{k}_i) \tag{52}$$

and that it is invariant under cobordisms representing continuous deformations of the map $\tilde{U}$ and the choice of x_0. [In particular, the cobordism theory also implies that the invariant cannot change in fine-tuned (i.e., not generic) situations where the pre-images are not point-like or where several point-like pre-images with the same orientation are brought on top of one another.] A pair of pre-images with opposite orientation (i.e., one being "covered" and the other "uncovered") can pairwise annihilate, while keeping the right-hand side of Eq. (51) invariant.

The relation in Eq. (51) is particularly useful when used to study Weyl points in the spectrum of $\tilde{U}(\mathbf{k})$. The eigenvalues of the special unitary in Eq. (39) are $\mathrm{e}^{\pm i\theta}$ where $n_0 = \cos\theta$ with $\theta \in [0, \pi]$. Note that the eigenvalues become degenerate (i.e., the bands of $\tilde{U}(\mathbf{k})$ exhibit a Weyl point) only when $\theta = 0$ [corresponding to the "north pole" of the 3-sphere] or $\theta = \pi$ [the "south pole" of the 3-sphere]. To prove the relation between $w_{3\mathrm{D}}$ and $C_{\mathrm{FS}(\mu)}$, we now study the relation between $w_{3\mathrm{D}}$ and the Weyl points of $\tilde{U}(\mathbf{k})$, whence Eq. (38) will be seen as a straightforward consequence.

The described band nodes are generically Weyl points, and they come in four species: They could occur at quasi-energy 0 or π, and they can carry left-handed or right-handed chirality. We indicate the number of these four species of Weyl points as N^0_{L}, N^0_{R}, N^π_{L} and N^π_{R}. It is detailed in Ref. 6 that positive (negative) coverings of $\pm\mathbb{1} \in \mathsf{SU}(2)$ produce right-handed (left-handed) Weyl points at the corresponding energy and $\mathbf{k}$, i.e., that $N^{0,\pi}_{L/R}$ correspond to

$\nu_\pm(\pm\mathbb{1})$. [The fact that a right-handed and left-handed Weyl point can pairwise annihilate is seen as a consequence of the index formula (52).] Since both $\pm\mathbb{1} \in \mathsf{SU}(2)$ have to be wrapped $w_{\rm 3D}$ times, it follows from Eq. (51) that

$$w_{\rm 3D} = N_{\rm R}^0 - N_{\rm L}^0 = N_{\rm R}^\pi - N_{\rm L}^\pi. \tag{53}$$

In other words, non-vanishing $w_{\rm 3D}$ implies an imbalance between right-handed and left-handed Fermions (i.e., violating the Nielsen-Ninomiya theorem[6,8]) both at quasi-energy 0 and π. It is well known (and confirmed by explicit calculation of a linearized model around the degeneracies) that right-/left-handed Weyl points inject/remove one qunatum of Berry curvature flow into the higher (quasi-)energy band. It therefore follows from the Weyl-point imbalance in two-band models with vanishing $w_{\rm 1D}$ that Eq. (38) is valid for all μ with non-degenerate Fermi surfaces (i.e., when the Chern number on the right-hand side can be meaningfully computed). We can thus interpret the right-hand side of Eq. (38) as the *flow* of Berry curvature in the quasi-energy direction. For example, for the band with quasi-energy $0 < \varepsilon_{\mathbf{k}} < \pi$, the right-handed (left-handed) Weyl points at $\varepsilon = 0$ act as sources (sinks) of quanta of Berry curvature, while the right-handed (left-handed) Weyl points at $\varepsilon = \pi$ instead act as sinks (sources) of quanta of Berry curvature.

Since Weyl points only occur at a discrete set of energies [namely at $\mu = 0$ and $\mu = \pi$ for $\tilde{U}(\mathbf{k}) \in \mathsf{SU}(2)$], one can use continuity to *define* the flow of Chern number on the right-hand side of Eq. (38) for arbitrary quasi-energy μ – including those coinciding with the Weyl point quasi-energy. Furthermore, because of the topological nature of the integrals on both sides of Eq. (38), the presented result generalizes to any two-band unitary map $U : \mathrm{BZ} \to \mathsf{U}(2)$ with vanishing $w_{\rm 1D}$, which are obtained from the explicitly discussed $\mathsf{SU}(2)$ models by a continuous deformation.

C. Relating $w_{\rm 3D}$ to Chern number in models with arbitrarily many bands and with $w_{\rm 1D} = 0$

The generalization of the relation in Eq. (38) to models with a larger number of bands follows directly by combining the (*i*) topological nature of integrals on both sides of the equation, (*ii*) additivity of both integrals under direct sum of two unitary maps $U_1 \oplus U_2$, and (*iii*) the existence of a continuous deformation (homotopy) between any pair of models with the same number of bands and with the same topological invariant $w_{\rm 3D} \in \pi[T^3, \mathsf{SU}(N)] = \mathbb{Z}$. In particular, it is important that the formula for $w_{\rm 3D}$ is *identical* for unitary operators with an arbitrary number $N \geq 2$ of bands.

To proceed, first note that two-band models can attain *arbitrary* $w_{\rm 3D} \in \mathbb{Z}$. For example, the special unitary in Eq. (39) with (before normalization)

$$\begin{aligned} n_0^W(\mathbf{k}) &= (\cos k_x + \cos k_y + \cos k_z - 2), \\ n_x^W(\mathbf{k}) &= \mathrm{Re}[(\sin k_x + \mathrm{i} \sin k_y)^W], \\ n_y^W(\mathbf{k}) &= \mathrm{Im}[(\sin k_x + \mathrm{i} \sin k_y)^W], \\ n_z^W(\mathbf{k}) &= \sin k_z, \end{aligned} \tag{54}$$

and with $W \in \mathbb{Z}$ has $w_{\rm 3D} = W$. We will introduce the notation for this particular model,

$$\frac{1}{||n^W(\mathbf{k})||^2} \left\{ n_0^W(\mathbf{k})\mathbb{1} - \mathrm{i}[n_x^W(\mathbf{k})\sigma_x + n_y^W(\mathbf{k})\sigma_y + n_z^W(\mathbf{k})\sigma_z] \right\} \equiv \tilde{U}^W(\mathbf{k}). \tag{55}$$

To proceed, let us investigate the validity of the equivalence in Eq. (38) for the special class of N-band models

$$\tilde{U}^{\rm W}(\mathbf{k}) \oplus \mathrm{e}^{\mathrm{i}\varepsilon_1} \oplus \ldots \oplus \mathrm{e}^{\mathrm{i}\varepsilon_{N-2}} \equiv \tilde{U}_N^W(\mathbf{k}) \tag{56}$$

where $\{\varepsilon_i\}_{i=1}^{N-2}$ are distinct and $\mathbf{k}$-independent phases (i.e., quasi-energies) subject to $\sum_i \varepsilon_i = 0$. We also require each of these phases to be different from 0 and π. On the one hand, we use the additivity of $w_{\rm 3D}$ for direct sum of unitaries, combined with the fact that $\mathbf{k}$-independent one-band unitaries have vanishing $w_{\rm 3D}$, to show that the N-band model in Eq. (56) has $w_{\rm 3D} = W$. On the other hand, the Chern number is also additive under direct sum of models, and contains a contribution from the two-band unitary $\tilde{U}^W(\mathbf{k})$ (for which it was proved in Supplementary Note 4.B. that $w_{\rm 3D} = C_{\mathrm{FS}(\mu)}$) plus the contributions of the single-band models (which for $\mu \neq \varepsilon_i$ do not form Fermi surface, and therefore their Chern number $C_{\mathrm{FS}(\mu)}$ trivially vanishes). Therefore, Eq. (38) is valid for models in Eq. (56) for all quasi-energies except $\mu \in \{0, \pi, \varepsilon_1, \ldots, \varepsilon_{N-2}\}$. Since this is a set of measure zero, we *define* the flow of Berry curvature $C_{\mathrm{FS}(\mu)}$ at these quasi-energies by continuity from the values where it is well-defined.

We now generalize the result in Eq. (38) to *arbitrary* N-band models $\tilde{U} : \mathrm{BZ} \to \mathsf{SU}(N)$. Recall that equivalence classes of such maps under continuous deformations are fully determined by the homotopy set $\pi[T^3, \mathsf{SU}(N)] \cong \mathbb{Z}$, with the corresponding class determined by the value of $w_{\rm 3D}$. Starting with an arbitrary N-band special unitary map

$\tilde{U}(\mathbf{k})$ that carries $w_{3D} = W$, it follows from homotopy theory that there is a continuous deformation into $\tilde{U}_N^W(\mathbf{k})$. The continuous deformation preserves w_{3D} by definition. Furthermore, such a continuous deformation must also preserve the Chern number at each energy. It is therefore a trivial consequence that Eq. (38) is valid for arbitrary special unitary models with arbitrarily many bands. By considering further continuous deformations, our proof trivially extends to all N-band models $U : \mathsf{BZ} \mapsto \mathsf{U}(N)$ that have $w_{1D} = 0$.

D. Relating w_{3D} to Chern number in models with arbitrary w_{1D}

The generalization of the relation in Eq. (38) to models with non-trivial w_{1D} is achieved through similar logic as exercised in the previous subsection when generalizing to arbitrarily many bands. Namely, we first prove the relation for specially crafted simple models – a representative model for each homotopy equivalence class – and then we argue (based on the invariant nature of the invariants under continuous deformations and on their additivity under direct sum of models) that the correspondence generalizes to arbitrary unitary models.

Recall that the set of homotopy classes of unitary models in 3D are $\pi[T^3, \mathsf{U}(N)] = \mathbb{Z}^4$ for any $N \geq 2$, where the four integer invariants correspond to winding numbers w_{3D} and $(w_{1D,x}, w_{1D,y}, w_{1D,z}) \equiv \mathbf{w}_{1D}$. We therefore consider the following $\mathsf{U}(3)$ models (a representative for each homotopy class),

$$U^{(W,\mathbf{w})}(\mathbf{k}) = \tilde{U}^W(\mathbf{k}) \oplus \mathrm{e}^{\mathrm{i}\mathbf{k}\cdot\mathbf{w}}, \tag{57}$$

for which it is easily checked that $w_{3D} = W$ and $\mathbf{w}_{1D} = \mathbf{w}$. Concerning the Chern number, we already argued in the previous subsections that $W = C_{\mathrm{FS}(\mu)}$ for the two-band part $\tilde{U}^W(\mathbf{k})$, while the one-band part $\mathrm{e}^{\mathrm{i}\mathbf{k}\cdot\mathbf{w}}$ manifestly carries zero Chern number as it encodes a constant (i.e., $\mathbf{k}$-independent) eigenstate $(0, 0, 1)$. Therefore, one readily finds that Eq. (38) is valid for the models in Eq. (57). Because of the continuous nature of the integrals defining $\mathbf{w}_{1D}$, W_{3D} and $C_{\mathrm{FS}(\mu)}$, the relation is readily generalized to *arbitrary* 3-band models $U : \mathsf{BZ} \to \mathsf{U}(3)$.

To generalize to models with $N \geq 3$ bands, one can consider [in analogy with Eq. (56) of Supplementary Note 4.C.] a direct-sum composition of the representative model in Eq. (57) with $N-3$ one-band models corresponding to constant quasi-energies. The proof of Eq. (38) for arbitrary $\mathsf{U}(N)$ model with $N \geq 3$ is then trivial to complete. Finally, the generalization to $N = 2$ model $U^{\text{2-band}}(\mathbf{k})$ is obtained similarly, with a single $\mathbf{k}$-independent band instead added to the two-band model itself [rather than to the homotopy representative in Eq. (57)]. Assuming that $U^{\text{2-band}}(\mathbf{k})$ carries $w_{3D} = W$ and $\mathbf{w}_{1D} = \mathbf{w}$, there is a homotopy equivalence

$$U^{\text{2-band}}(\mathbf{k}) \oplus \mathrm{e}^{\mathrm{i}\varepsilon_1} \sim \tilde{U}^W(\mathbf{k}) \oplus \mathrm{e}^{\mathrm{i}\mathbf{k}\cdot\mathbf{w}}, \tag{58}$$

whence the relation in Eq. (38) follows for the two-band model $U^{\text{2-band}}(\mathbf{k})$ from the additivity of invariants in direct-sum models. Therefore, we complete the proof of the equivalence $w_{3D} = C_{\mathrm{FS}(\mu)}$ (for arbitrary quasi-energy μ) for arbitrary unitary map $U : \mathsf{BZ} \to \mathsf{U}(N)$ with arbitrary $N \geq 2$. Finally, by recalling the continuity arguments of Supplementary Note 4.A., the equivalence generalizes to non-Hermitian point-gapped Hamiltonians whenever the Chern number is well-defined (i.e., whenever the Fermi surface is not intersected by an exceptional degeneracy).

SUPPLEMENTARY NOTE 5: NON-HERMITIAN TERMS IN ELECTRONIC SYSTEMS

In this section, we discuss scenarios, in which the specific structure of the self-energy discussed in the main text could arise in a condensed matter setting. In general, the emergence of non-Hermitian terms in electronic systems is best understood in the language of Green's functions, where the terms arise through a complex self-energy. The non-interacting Green's function is given by

$$G^{(0)}(\omega) = (\mathrm{i}\omega - H)^{-1}, \tag{59}$$

with H being the free Hamiltonian of the (electronic) system. This Green's function describes infinitely long-lived excitations at the eigenenergies of H. Interactions can be incorporated into the full Green's function introducing the self-energy $\Sigma(\omega)$,

$$G(\omega) = \{[G^{(0)}(\omega)]^{-1} - \Sigma(\omega)\}^{-1} = [\mathrm{i}\omega - H - \Sigma(\omega)]^{-1} \equiv [\mathrm{i}\omega - H_{\mathrm{eff}}]^{-1}, \tag{60}$$

where in the last step, we have dropped the ω dependence of Σ. If the self-energy is complex, for example due to finite quasi-particle lifetimes, the effective Hamiltonian of the system H_{eff} is non-Hermitian.

A. Non-Hermitian terms due to coupling to short-lived electronic excitations

One scenario to obtain a complex self-energy as discussed in the main text, is to consider an additional orbital—for concreteness, we call it an f electron—with no dispersion and a short lifetime $1/\Gamma$, which couples to the s, p orbitals of the topological insulator. The (effective) Hamiltonian of this additional f orbital is

$$H_{\mathrm{f}} = \mu_{\mathrm{f}} c_{\mathrm{f}}^{\dagger} c_{\mathrm{f}} + i\Gamma c_{\mathrm{f}}^{\dagger} c_{\mathrm{f}}. \tag{61}$$

Considering a hopping Hamiltonian between the s, p, and f electrons of the form

$$H_{\mathrm{sp-f}} = t_{\mathrm{s}} c_{\mathrm{s}}^{\dagger} c_{\mathrm{f}} + t_{\mathrm{p}} c_{\mathrm{p}}^{\dagger} c_{\mathrm{f}}, \tag{62}$$

we can find the full Hamiltonian in block form as

$$H = \begin{pmatrix} H_{\mathrm{sp}} & H_{\mathrm{sp-f}} \\ H_{\mathrm{sp-f}}^{\dagger} & H_{\mathrm{f}} \end{pmatrix}. \tag{63}$$

Since the full Green's function is defined as

$$(i\omega - H)\, G(\omega) = \mathbb{1}, \tag{64}$$

we obtain

$$\begin{pmatrix} i\omega - H_{\mathrm{sp}} & -H_{\mathrm{sp-f}} \\ -H_{\mathrm{sp-f}}^{\dagger} & i\omega - H_{\mathrm{f}} \end{pmatrix} \begin{pmatrix} G_{\mathrm{sp}}(\omega) & G_{\mathrm{sp-f}}(\omega) \\ G_{\mathrm{f-sp}}(\omega) & G_{\mathrm{f}}(\omega) \end{pmatrix} = \mathbb{1}. \tag{65}$$

Using

$$G_{\mathrm{f-sp}}(\omega) = (i\omega - H_{\mathrm{f}})^{-1} H_{\mathrm{sp-f}}^{\dagger} G_{\mathrm{sp}}(\omega), \tag{66}$$

we can rewrite

$$\begin{aligned} (i\omega - H_{\mathrm{sp}})\, G_{\mathrm{sp}}(\omega) - H_{\mathrm{sp-f}} G_{\mathrm{f-sp}}(\omega) &= (i\omega - H_{\mathrm{sp}})\, G_{\mathrm{sp}}(\omega) - H_{\mathrm{sp-f}}\, (i\omega - H_{\mathrm{f}})^{-1} H_{\mathrm{sp-f}}^{\dagger} G_{\mathrm{sp}}(\omega) \\ &= (i\omega - H_{\mathrm{sp}} - \Sigma(\omega))\, G_{\mathrm{sp}}(\omega) = 1. \end{aligned} \tag{67}$$

This gives a correction to the s- and p-electron self energy

$$\Sigma(\omega) = (t_{\mathrm{s}}\ t_{\mathrm{p}})^{T} (i\omega - H_{\mathrm{f}})^{-1} (t_{\mathrm{s}}\ t_{p}) = \frac{1}{i\omega - \mu_{\mathrm{f}} - i\Gamma} \begin{pmatrix} t_{\mathrm{s}}^2 & t_{\mathrm{s}} t_{\mathrm{p}} \\ t_{\mathrm{s}} t_{\mathrm{p}} & t_{\mathrm{p}}^2 \end{pmatrix}. \tag{68}$$

To lowest order in ω, the self energy thus reads

$$\Sigma \approx \begin{pmatrix} \frac{\mathrm{i} t_{\mathrm{s}}^2}{\Gamma - \mathrm{i}\mu_{\mathrm{f}}} & \frac{\mathrm{i} t_{\mathrm{s}} t_{\mathrm{p}}}{\Gamma - \mathrm{i}\mu_{\mathrm{f}}} \\ \frac{\mathrm{i} t_{\mathrm{s}} t_{\mathrm{p}}}{\Gamma - \mathrm{i}\mu_{\mathrm{f}}} & \frac{\mathrm{i} t_{\mathrm{p}}^2}{\Gamma - \mathrm{i}\mu_{\mathrm{f}}} \end{pmatrix} = \frac{\mathrm{i}}{2} \frac{t_{\mathrm{s}}^2 + t_{\mathrm{p}}^2}{\Gamma - \mathrm{i}\mu_{\mathrm{f}}} \tau_0 + \mathrm{i} \frac{t_{\mathrm{s}} t_{\mathrm{p}}}{\Gamma - \mathrm{i}\mu_{\mathrm{f}}} \tau_x + \frac{\mathrm{i}}{2} \frac{t_{\mathrm{s}}^2 - t_{\mathrm{p}}^2}{\Gamma - \mathrm{i}\mu_{\mathrm{f}}} \tau_z, \tag{69}$$

which adds to the effective Hamiltonian of s and p electrons. Considering $t_{\mathrm{s}} \approx t_{\mathrm{p}} \equiv t_{\mathrm{f}}$, the contributions proportional to τ_z vanish. Including spins of s, p orbitals, we obtain the self-energy

$$\Sigma = \mathrm{i} \frac{t_{\mathrm{f}}^2}{\Gamma - \mathrm{i}\mu_{\mathrm{f}}} (\tau_0 + \tau_x) \sigma_0. \tag{70}$$

If the f electron sits close to the chemical potential, in other words $\mu_{\mathrm{f}} \ll \Gamma$, the non-Hermitian term dominates in Eq. (70) and gives rise to the physics discussed in the main text.

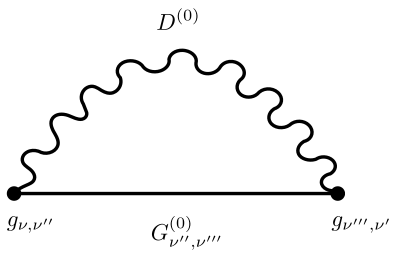

Supplementary Figure 5. Lowest-order Feynman diagram of the electron self-energy from the electron-phonon coupling.

B. Non-Hermitian terms due to electron-phonon scattering

As an alternative scenario, we consider the scattering of electrons and phonons, one of the most fundamental interactions in solids. For simplicity, we describe this coupling with a toy model, the Holstein Hamiltonian, which in second quantization reads

$$\begin{aligned} H &= H_{\mathrm{el}} + H_{\mathrm{ph}} + H_{\mathrm{el-ph}} \\ &= \sum_{\mathbf{k}} c^\dagger_{\mathbf{k},\nu} \left(\mathbf{k}\cdot\Gamma - \mu\right)_{\nu,\nu'} c_{\mathbf{k},\nu'} + \omega_0 \sum_{\mathbf{q}} \left(b^\dagger_{\mathbf{q}} b_{\mathbf{q}} + \frac{1}{2}\right) + \sum_{\mathbf{k},\mathbf{q},\nu,\nu'} \frac{g_{\nu,\nu'}}{\sqrt{N}} \left(b^\dagger_{\mathbf{q}} + b_{-\mathbf{q}}\right) c^\dagger_{\mathbf{k}+\mathbf{q},\nu} c_{\mathbf{k},\nu'}. \end{aligned} \tag{71}$$

Here, we consider non-interacting electrons with a 3D Dirac dispersion $\mathbf{k}\cdot\Gamma$, with $\Gamma_i = \tau_x\sigma_i$, and model the phonons as a single branch of Einstein phonons of frequency ω_0 and momentum $\mathbf{q}$. The coupling strength $g_{\nu,\nu'}$ is taken to be small and momentum independent but we allow electrons to scatter from ν to ν'. Here and in the following, we use ν as a combined index of orbital and spin.

The interaction with the phonons leads to a non-trivial self-energy in the electron Green's function

$$G(\mathbf{k},\mathrm{i}\omega_m)^{-1}_{\nu,\nu'} = G^{(0)}(\mathbf{k},\mathrm{i}\omega_m)^{-1}_{\nu,\nu'} + \Sigma_{\mathrm{el-ph}}(\mathbf{k},\mathrm{i}\omega_m)_{\nu,\nu'} \tag{72}$$

with the bare (non-interacting) electron Green's function given by

$$G^{(0)}(\mathbf{k},\mathrm{i}\omega_m)_{\nu,\nu'} = (\mathrm{i}\omega_m - H_{\mathrm{el}})^{-1}_{\nu,\nu'} \tag{73}$$

and $\omega_m = (2m+1)\pi T$ (m an integer) are the fermionic Matsubara frequencies. In the following, we use a lowest-order approximation to the electron-phonon problem[9], since we are only interested in the *structure* of the resulting self-energy. The self-energy can be calculated as

$$\Sigma_{\mathrm{el-ph}}(\mathbf{k},\mathrm{i}\omega_m)_{\nu,\nu'} = -\frac{1}{N\beta}\sum_{\mathbf{k}',m'}\sum_{\nu'',\nu'''} g_{\nu,\nu''} G(\mathbf{k}',\mathrm{i}\omega_{m'})_{\nu'',\nu'''} g_{\nu''',\nu'} D(\mathbf{q},\mathrm{i}(\omega_m-\omega_{m'})), \tag{74}$$

where we have relied on Migdal-Eliashberg theory to neglect vertex corrections[10,11]. This is valid even for the case of Dirac materials[12], but only correct in the weak-coupling regime $g/\omega_0 \ll 1$[13,14]. It is also customary to replace the full electron and phonon Green's functions in Eq. (74) to lowest order with the bare Green's function in Eq. (73) and the bare phonon Green's function[10,11]

$$D^{(0)}(\mathbf{q},\mathrm{i}\Omega_n) = \left[\omega_0^2 + \Omega_n^2\right]^{-1}, \tag{75}$$

with $\Omega_n = 2n\pi T$ the bosonic Matsubara frequencies. This corresponds to the processes of emitting and subsequently absorbing a phonon with momentum $\mathbf{q}$ ($-\mathbf{q}$), see Supplementary Fig. 5.

The electron self-energy thus simplifies to

$$\Sigma_{\mathrm{el-ph}}(\mathbf{k},\mathrm{i}\omega_m)_{\nu,\nu'} = -\frac{1}{N\beta}\sum_{\mathbf{k}',m'}\left[g\frac{(\mathrm{i}\omega_{m'}+\mu)\mathbb{1} + \mathbf{k}'\cdot\Gamma}{(\mathrm{i}\omega_{m'}+\mu)^2 - |\mathbf{k}'|^2} g \frac{1}{[(\omega_0^2 + (\omega_m-\omega_{m'})^2)]}\right]_{\nu,\nu'}, \tag{76}$$

where, for simplicity, we have neglected the matrix indices of $g_{\nu,\nu'}$ and the element ν,ν' is taken after matrix multiplication. The term linear in k' vanishes upon integration, such that, performing the summation over the Matsubara frequencies, we find

$$\begin{aligned}\frac{1}{\beta}\sum_{m'}\frac{(\mathrm{i}\omega_{m'}+\mu)}{(\mathrm{i}\omega_{m'}+\mu)^2-|\mathbf{k}'|^2}\frac{1}{[(\omega_0^2+(\omega_m-\omega_{m'})^2)]} &= -\frac{1}{2}\frac{n_{\mathrm{F}}(-|\mathbf{k}'|-\mu)}{(\mathrm{i}\omega_m+|\mathbf{k}'|+\mu)^2-\omega_0^2}-\frac{1}{2}\frac{n_{\mathrm{F}}(|\mathbf{k}'|-\mu)}{(\mathrm{i}\omega_m-|\mathbf{k}'|+\mu)^2-\omega_0^2}\\ &+\frac{1}{2}\frac{(\mathrm{i}\omega_m+\mu+\omega_0)n_{\mathrm{B}}(\omega_0)}{\omega_0(\mathrm{i}\omega_m-|\mathbf{k}'|+\mu+\omega_0)(\mathrm{i}\omega_m+|\mathbf{k}'|+\mu+\omega_0)}\\ &+\frac{1}{2}\frac{(\mathrm{i}\omega_m+\mu-\omega_0)(1+n_{\mathrm{B}}(\omega_0))}{\omega_0(\mathrm{i}\omega_m-|\mathbf{k}'|+\mu-\omega_0)(\mathrm{i}\omega_m+|\mathbf{k}'|+\mu-\omega_0)}\\ &= F_{\mathrm{F}}(\mathbf{k}',\mathrm{i}\omega_m)+F_{\mathrm{B}}(\mathbf{k}',\mathrm{i}\omega_m).\end{aligned} \tag{77}$$

To continue, we have separated the first two fermionic and the last two bosonic terms, and $n_{\mathrm{F}}(x)$, $n_{\mathrm{B}}(x)$ are the fermion and boson distribution functions, respectively. We thus find the self-energy

$$\Sigma_{\mathrm{el-ph}}(\mathbf{k},\mathrm{i}\omega_m)_{\nu,\nu'} = -\frac{1}{N}\sum_{\mathbf{k}'}(F_{\mathrm{F}}(\mathbf{k}',\mathrm{i}\omega_m)+F_{\mathrm{B}}(\mathbf{k}',\mathrm{i}\omega_m))(g^2)_{\nu,\nu'}, \tag{78}$$

where $(g^2)_{\nu,\nu'}=\sum_{\nu''}g_{\nu,\nu''}g_{\nu'',\nu'}$. In the following, we perform the remaining integration in $\mathbf{k}'$:

$$\begin{aligned}-\frac{1}{N}\sum_{\mathbf{k}'}F_{\mathrm{F}}(\mathbf{k}',\mathrm{i}\omega_m) &= \frac{1}{4\pi^2}\int_0^\infty dk'k'^2\left[\frac{n_{\mathrm{F}}(-k'-\mu)}{(\mathrm{i}\omega_m+k'+\mu)^2-\omega_0^2}+\frac{n_{\mathrm{F}}(k'-\mu)}{(\mathrm{i}\omega_m-k'+\mu)^2-\omega_0^2}\right]\\ &= \frac{1}{4\pi^2}\left[\int_{-\infty}^{-\mu}d\hat{k}\frac{(\hat{k}+\mu)^2n_{\mathrm{F}}(\hat{k})}{(\mathrm{i}\omega_m-\hat{k})^2-\omega_0^2}+\int_{-\mu}^{\infty}d\hat{k}\frac{(\hat{k}+\mu)^2n_{\mathrm{F}}(\hat{k})}{(\mathrm{i}\omega_m-\hat{k})^2-\omega_0^2}\right]\\ &= \frac{1}{4\pi^2}\int_{-\infty}^{\infty}d\hat{k}\frac{(\hat{k}+\mu)^2n_{\mathrm{F}}(\hat{k})}{(\mathrm{i}\omega_m-\hat{k})^2-\omega_0^2},\end{aligned} \tag{79}$$

where, in the second line, we substituted $\hat{k}=-k'-\mu$ in the first and $\hat{k}=k'-\mu$ in the second term. Performing the analytic continuation $\hat{k}\to z$ yields

$$\frac{1}{4\pi^2}\int_{-\infty}^{\infty}f(z)dz=\frac{1}{4\pi^2}\int_{-\infty}^{\infty}dz\frac{(z+\mu)^2n_{\mathrm{F}}(z)}{(z-\mathrm{i}\omega_m+\omega_0)(z-\mathrm{i}\omega_m-\omega_0)}, \tag{80}$$

where $f(z)$ has two poles $z_{1,2}=\mathrm{i}\omega_m\pm\omega_0$ in the upper half of the complex plane. Choosing an integration contour $\mathcal{C}$ of radius R in the upper half surrounding the poles allows to evaluate the integral with the residue theorem. As the arc in the complex plane vanishes in the limit $R\to\infty$, the fermionic term yields

$$-\frac{1}{N}\sum_{\mathbf{k}'}F_{\mathrm{F}}(\mathbf{k}',\mathrm{i}\omega_m)=-\frac{\mathrm{i}}{4\pi\omega_0}\left[(\mathrm{i}\omega_m+\mu-\omega_0)^2n_{\mathrm{F}}(\mathrm{i}\omega_m-\omega_0)-(\mathrm{i}\omega_m+\mu+\omega_0)^2n_{\mathrm{F}}(\mathrm{i}\omega_m+\omega_0)\right]. \tag{81}$$

The bosonic integral

$$-\frac{1}{N}\sum_{\mathbf{k}'}F_{\mathrm{B}}(\mathbf{k}',\mathrm{i}\omega_m)=-\frac{1}{\omega_0}\frac{1}{4\pi^2}\int_0^\infty dk'k'^2\left[\frac{(\mathrm{i}\omega_m+\mu+\omega_0)n_{\mathrm{B}}(\omega_0)}{(\mathrm{i}\omega_m+\mu+\omega_0)^2-k'^2}+\frac{(\mathrm{i}\omega_m+\mu-\omega_0)(1+n_{\mathrm{B}}(\omega_0))}{(\mathrm{i}\omega_m+\mu-\omega_0)^2-k'^2}\right] \tag{82}$$

is formally divergent due to large and intermediate momentum states. We therefore introduce a regularization parameter λ, so that singularities appear solely in this quantity. Correspondingly, we limit the momentum integral to the region $|\mathbf{k}'|<\lambda$, in which the description with the effective Dirac Hamiltonian remains valid

$$\begin{aligned}-\frac{1}{N}\sum_{\mathbf{k}'}F_{\mathrm{B}}(\mathbf{k}',\mathrm{i}\omega_m) &= -\frac{1}{4\pi^2\omega_0}\int_0^\lambda dk'k'^2\left[\frac{(\mathrm{i}\omega_m+\mu+\omega_0)n_{\mathrm{B}}(\omega_0)}{(\mathrm{i}\omega_m+\mu+\omega_0)^2-k'^2}+\frac{(\mathrm{i}\omega_m+\mu-\omega_0)(1+n_{\mathrm{B}}(\omega_0))}{(\mathrm{i}\omega_m+\mu-\omega_0)^2-k'^2}\right]\\ &= -\frac{1}{4\pi^2\omega_0}\Bigg[n_{\mathrm{B}}(\omega_0)(\mathrm{i}\omega_m+\mu+\omega_0)^2\operatorname{artanh}\left(\frac{\lambda}{\mathrm{i}\omega_m+\mu+\omega_0}\right)-n_{\mathrm{B}}(\omega_0)(\mathrm{i}\omega_m+\mu+\omega_0)\lambda\\ &\quad+(1+n_{\mathrm{B}}(\omega_0))(\mathrm{i}\omega_m+\mu-\omega_0)^2\operatorname{artanh}\left(\frac{\lambda}{\mathrm{i}\omega_m+\mu-\omega_0}\right)-(1+n_{\mathrm{B}}(\omega_0))(\mathrm{i}\omega_m+\mu-\omega_0)\lambda\Bigg].\end{aligned} \tag{83}$$

The combination of both terms in the self-energy yields

$$\begin{aligned}\Sigma_{\mathrm{el-ph}}(\mathbf{k},\mathrm{i}\omega_m)_{\nu,\nu'} &= -\frac{1}{N}\sum_{\mathbf{k}'}(F_{\mathrm{F}}(\mathbf{k}',\mathrm{i}\omega_m)+F_{\mathrm{B}}(\mathbf{k}',\mathrm{i}\omega_m))(g^2)_{\nu,\nu'} \\ &= \Phi(\mathrm{i}\omega_m)(g^2)_{\nu,\nu'},\end{aligned} \tag{84}$$

where we combined the integrals in the function Φ. Finally, we can analyze the matrix structure of the self-energy. Since the electron-phonon scattering can in principle scatter between s and p orbitals but is spin preserving, we parametrize $g_{\nu,\nu'} = g_\rho \tau_\rho \sigma_0$, where $\rho = 0, x, y, z$, τ_ρ are Pauli matrices, and σ_0 is the 2×2 identity. This yields

$$(g^2)_{\nu,\nu'} = \Big[\sum_\rho g_\rho^2 \tau_0\sigma_0 + 2\sum_a g_0 g_a \tau_a \sigma_0 + \mathrm{i}\sum_{a,b} g_a g_b \epsilon_{abc}\tau_c\sigma_0\Big]_{\nu\nu'}, \tag{85}$$

where $a = x, y, z$. Note that for $g_x \neq 0$, the phonon needs to break inversion symmetry, while $g_y \equiv 0$ for time-reversal-symmetric scattering. Finally, if we assume $g_x \gg g_z$, we obtain

$$gg \approx (g_0^2 + g_x^2)\tau_0\sigma_0 + 2g_0 g_x \tau_x \sigma_0, \tag{86}$$

which results in a self-energy of the form

$$\Sigma_{\mathrm{el-ph}}(\mathrm{i}\omega_m) = \Phi(\mathrm{i}\omega_m)\left((g_0^2 + g_x^2)\tau_0\sigma_0 + 2g_0 g_x \tau_x\sigma_0\right), \tag{87}$$

where we removed the momentum argument, since the right hand side does not contain a **k**-dependence.

At small ω_m, $\Phi(\mathrm{i}\omega_m) \approx \Phi(0)$, we can investigate the influence of the cutoff λ. Being the dominant scale in the problem, we take $\lambda \gg \omega_0$, where $\omega_0 > \mu$. This allows to expand the hyperbolic tangent in Eq. (83),

$$\operatorname{artanh}\left(\frac{\lambda}{\mu+\omega_0}\right) \approx -\mathrm{i}\frac{\pi}{2}, \qquad \frac{\lambda}{\mu+\omega_0} \gg 1, \tag{88}$$

$$\operatorname{artanh}\left(\frac{\lambda}{\mu-\omega_0}\right) \approx \mathrm{i}\frac{\pi}{2}, \qquad \frac{\lambda}{\mu-\omega_0} \ll -1, \tag{89}$$

which yields

$$\begin{aligned}\Phi(0) &= -\frac{1}{N}\sum_{\mathbf{k}'}(F_{\mathrm{F}}(\mathbf{k}',0)+F_{\mathrm{B}}(\mathbf{k}',0)) \\ &= \frac{\mathrm{i}}{4\pi\omega_0}\left[(\mu+\omega_0)^2 n_{\mathrm{F}}(\omega_0) - (\mu-\omega_0)^2(1-n_{\mathrm{F}}(\omega_0))\right] \\ &\quad + \frac{\mathrm{i}}{8\pi\omega_0}\left(4\mu\omega_0 n_{\mathrm{B}}(\omega_0) - (\mu-\omega_0)^2\right) + \frac{\lambda}{4\pi^2\omega_0}\left(2\mu n_{\mathrm{B}}(\omega_0) + \mu - \omega_0\right).\end{aligned} \tag{90}$$

The last term constitutes the real part of the self-energy, which can be parametrically small by a suitable combination of $\omega_0, \mu, n_{\mathrm{B}}(\omega_0)$. This means the imaginary part of the self-energy becomes dominant by a suitable choice of the phonon frequency ω_0 and the selective driving via $n_{\mathrm{B}}(\omega_0)$, while $\mu \ll \omega_0$. In such a setting, $\Phi(0) \approx \mathrm{i}\phi(0)$, yielding

$$\Sigma_{\mathrm{el-ph}}(0) = \mathrm{i}\beta\tau_0\sigma_0 + \mathrm{i}\delta\tau_x\sigma_0, \tag{91}$$

with $\beta = \phi(0)(g_0^2 + g_x^2)$ and $\delta = 2\phi(0)g_0 g_x$. The appearance of the term $\beta\tau_0\sigma_0$ shifts the point gap on the imaginary axis, without altering the properties discussed in the main body of the paper.

The result derived above is only valid for small electron-phonon couplings g/ω_0. For stronger couplings, the self-energy has to be calculated to higher orders in g or even self-consistently. Such a rather tedious calculation requires to take care of the branch cuts in (84). Importantly, the overall structure of the self-energy is preserved to the next order in the coupling constant.

SUPPLEMENTARY NOTE 6: DISCUSSION OF TWO BAND MODELS WITH NON-TRIVIAL w_{3D}

Two-band models often serve as minimal models describing the physics of topological phases in case of Hermitian Hamiltonians. Here we would like to discuss two specific examples of non-Hermitian two-band models with a point gap in their spectrum: non-Hermitian extensions of a Chern insulator and a non-Hermitian Weyl semimetal proposed in Ref. 3 and 15 and point out that these models are not easily regularizable to host a single exceptional point.

A. Non-Hermitian Chern insulator

First, consider a non-Hermitian variant of Chern insulator in 2D rotated in the complex plane, described by

$$H = \frac{(1+\cos k_x + \cos k_y)\sigma_z + \sin k_x \sigma_x + \sin k_y \sigma_y}{\sqrt{3 + 2\cos k_x + \cos(k_x - k_y) + 2\cos k_y + \cos(k_x + k_y)}} + \left(\frac{1}{2}e^{ik_z} - 1\right)\sigma_0. \tag{92}$$

This model possesses a point gap in the bulk spectrum, see Supplementary Fig. 6a. Even though the 3D winding number for this model is non-trivial $w_{3D} = 1$, open boundaries in z-direction yield the non-Hermitian skin effect, with occupations concentrating on one side of the system as shown in Supplementary Fig. 6b. For open boundaries in x and y-direction, the point gap regions are filled by edge states. The corresponding weak invariants $w_{1D,i=\{x,y,z\}}$ support this observation, being zero for x, y and one in z-direction. The appearance of the non-Hermitian skin effect, signalled by a non-trivial w_{1D} invariant, hence causes a collapse of the point gap. This disqualifies the model in Eq.(92) for hosting stable surface states like for instance exceptional points.

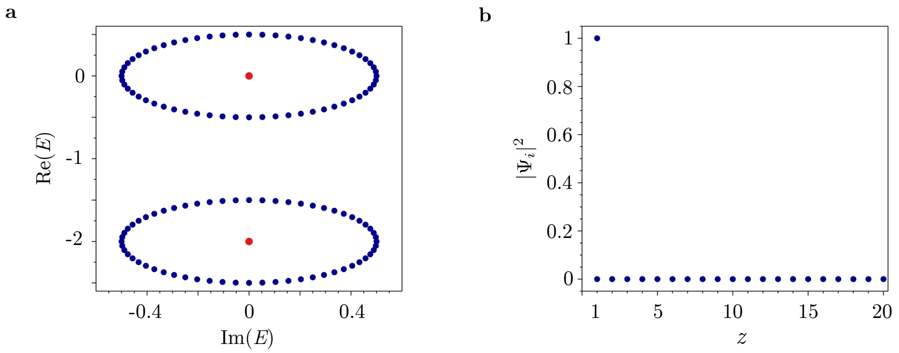

Supplementary Figure 6. **Energy spectra and localization properties of the non-Hermitian Chern insulator. a** Bulk spectrum of the non-Hermitian Chern insulator (blue) and open boundary condition spectrum in z direction (red) for a system of 20 layers. **b** The occupation of lattice sites for OBC in z direction for a system of 20 layers.

B. Non-Hermitian Weyl semimetal

The two-band model describing a dissipative Weyl semimetal with a point gap in the bulk spectrum has recently been proposed in Ref. 3 and 15. The non-Hermitian Hamiltonian of the model reads as follows

$$H_{\mathrm{NHWS}} = \mathrm{i}\left(h + \sum_{i\in\{x,y,z\}} \cos k_i\right)\sigma_0 + \sum_{i\in\{x,y,z\}} \sin k_i \sigma_i. \tag{93}$$

The determinant of this Hamiltonian is real at every **k**-point, therefore there are no 1D winding numbers, whereas the 3D winding number is non-trivial. The weak Chern numbers cannot be properly defined due to the occurrence of Weyl points at TRIMs.

The spectrum for open boundaries in the x direction features three analytically calculated eigenvalues at $\epsilon_{1,2,3} = i(h \pm 2), ih$ appearing in the point gap. Each of these states is 2N-fold degenerate, where N is the number of layers. The ellipses of states being present around the analytical solutions in Supplementary Fig. 7a are due to the numerical instability of the model. This resembles the infernal point discussed in the main section of the paper, as the surface

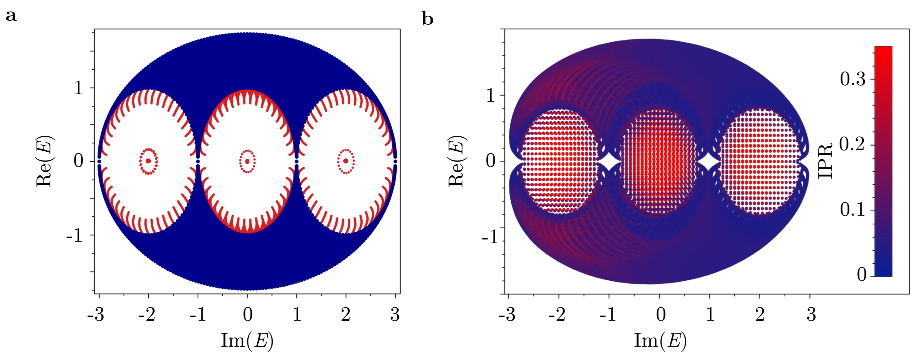

Supplementary Figure 7. **Energy spectrum in the complex plane for the non-Hermitian Weyl semimetal. a** Bulk energy spectrum (blue) and spectrum for open boundary conditions in x direction (red) for the model in Eq. (93) for $h = 0$ and 20 layers. The analytical solution shows eigenstates at the center of the point gaps. **b** Open boundary condition spectrum for the regularization term introduced in Eq. (94) ($B = 0.1$, 20 layers), resulting in a single surface band covering the point gaps, indicated in red-to-blue tones according to their inverse participation ratio (IPR).

state is fine-tuned rather than generic. Similar to the four-band case this surface state can be regularized along certain momentum directions by adding a small perturbation

$$H = H_{\text{NHWS}} + B\left[(1-\cos k_x)\,\sigma_y + (1-\cos k_y)\,\sigma_z + (1-\cos k_z)\,\sigma_x\right], \tag{94}$$

which yields a single sheet of eigenstates covering the OBC point gap as illustrated in Supplementary Fig. 7b. The above perturbation moves Weyl cones in the Brillouin zone, ensuring that they do not fall on the same momentum direction when considering open boundary conditions. In a two-band model, this is not possible to ensure with constant regularization terms for surface orientations along cardinal axes. Correspondingly, without a k-dependent perturbation, there is always a direction in momentum space along which the OBC system has an infernal point, with this direction being the connection of two Weyl points.

C. Effective theory at a spin-orbit coupled band edge

In the main text, we focus on how an ETI can arise near the critical Dirac point between a topological and a trivial insulator upon the addition of a suitable non-Hermitian coupling. The rational for considering this case is that the density of states is very low near such a Dirac point, and hence it is the most favorable situation for observing the physical consequences of the ETI point gap topology. However, point gaps with the same topology can also arise in other generic situations. One that corresponds to a reasonably small density of states is the band edge of a generic spin-orbit coupled band. To demonstrate this, let us analyse the behaviour of the ETI around $\mathbf{k} = (\pi,\pi,\pi)$ by projecting the Hamiltonian onto the states corresponding to the upper-bands. Diagonalizing the original Hamiltonian, Eq. (1) from the main text, $H(\pi,\pi,\pi)$ with $B = 0$ for simplicity, we obtain two eigenvalues $E_\pm = \pm\sqrt{(3+M-\delta)(3+M+\delta)}$ each doubly degenerate with corresponding eigenvectors given by the rows of the matrices V for the upper-band and W for the lower-energy band as follows

$$V = \frac{1}{\mathcal{N}_v}\begin{pmatrix} 0 & v & 0 & 1 \\ v & 0 & 1 & 0 \end{pmatrix}, \qquad W = \frac{1}{\mathcal{N}_w}\begin{pmatrix} 0 & w & 0 & 1 \\ w & 0 & 1 & 0 \end{pmatrix}, \tag{95}$$

where

$$\begin{aligned} v &= -\frac{i(-3-M+\sqrt{(3+M-\delta)(3+M+\delta)})}{\delta}, \\ w &= -\frac{i(3+M+\sqrt{(3+M-\delta)(3+M+\delta)})}{\delta} \end{aligned} \tag{96}$$

are normalization factors

$$\mathcal{N}_v = \sqrt{1 + \frac{(-3 - M + \sqrt{(3+M-\delta)(3+M+\delta)})^2}{\delta^2}}$$
$$\mathcal{N}_w = \sqrt{1 + \frac{(3 + M + \sqrt{(3+M-\delta)(3+M+\delta)})^2}{\delta^2}}. \quad (97)$$

We expand $H(\mathbf{k})$ around $\mathbf{k} = (\pi,\pi,\pi)$, shifting momentum $\mathbf{k} \to \mathbf{p} + (\pi,\pi,\pi)$ and keeping only the terms of the first order in $\mathbf{p}$. The linearized Hamiltonian reads as follows

$$h(\mathbf{p}) = (-3 - M)\tau_z\sigma_0 + i\delta\tau_x\sigma_0 - \lambda \sum_{j=x,y,z} p_j \tau_x \sigma_j. \quad (98)$$

We apply degenerate perturbation theory to first order in $\mathbf{p}$ and to first order in δ. The corrections to the energy in the upper-band are going to be the eigenvalues of the following matrix

$$h_{\text{eff}}(\mathbf{p}) = Vh(\mathbf{p})V^\dagger - \frac{Vh(\mathbf{p})W^\dagger W h(\mathbf{p})V^\dagger}{E_+ - E_-} = q_1\sigma_0 - iq_2\boldsymbol{\xi}\cdot\boldsymbol{\sigma} + \mathcal{O}(\mathbf{p}^2)\,, \quad (99)$$

where $\boldsymbol{\xi} = (-p_x, p_y, p_z)$, $q_1 = \sqrt{(3+M-\delta)(3+M+\delta)}$, and $q_2 = \delta\lambda/(3+M)$. The resulting linearized Hamiltonian in Eq.(99) is of the same form as the Hamiltonian in Eq. (93) for small $\mathbf{k}$, which has been studied earlier in Ref. 3. Thus, an ETI Hamiltonian arises generically at the band edge of a spin-orbit coupled band when subject to a suitable non-Hermitian perturbation. The fact that q_2 is linear in λ emphasizes the importance of spin-orbit coupling for this result.

[1] Gong, Z. *et al.* Topological phases of non-hermitian systems. *Phys. Rev. X* **8**, 031079 (2018). URL https://link.aps.org/doi/10.1103/PhysRevX.8.031079.

[2] Kawabata, K., Shiozaki, K., Ueda, M. & Sato, M. Symmetry and topology in non-hermitian physics. *Physical Review X* **9** (2019). URL http://dx.doi.org/10.1103/PhysRevX.9.041015.

[3] Terrier, F. & Kunst, F. K. Dissipative analog of four-dimensional quantum Hall physics. *arXiv e-prints* arXiv:2003.11042 (2020). URL https://ui.adsabs.harvard.edu/abs/2020arXiv200311042T.

[4] Demange, G. & Graefe, E.-M. Signatures of three coalescing eigenfunctions. *J. Phys. A: Math. Theor* **45**, 025303 (2012). URL http://dx.doi.org/10.1088/1751-8113/45/2/025303.

[5] Bessho, T. & Sato, M. Topological Duality in Floquet and Non-Hermitian Dynamical Anomalies: Extended Nielsen-Ninomiya Theorem and Chiral Magnetic Effect. *arXiv e-prints* arXiv:2006.04204 (2020). URL https://ui.adsabs.harvard.edu/abs/2020arXiv200604204B.

[6] Sun, X.-Q., Xiao, M., Bzdušek, T., Zhang, S.-C. & Fan, S. Three-dimensional chiral lattice fermion in floquet systems. *Phys. Rev. Lett.* **121**, 196401 (2018). URL https://link.aps.org/doi/10.1103/PhysRevLett.121.196401.

[7] Kennedy, R. Topological hopf-chern insulators and the hopf superconductor. *Phys. Rev. B* **94**, 035137 (2016). URL https://link.aps.org/doi/10.1103/PhysRevB.94.035137.

[8] Higashikawa, S., Nakagawa, M. & Ueda, M. Floquet chiral magnetic effect. *Phys. Rev. Lett.* **123**, 066403 (2019). URL https://link.aps.org/doi/10.1103/PhysRevLett.123.066403.

[9] Giustino, F. Electron-phonon interactions from first principles. *Rev. Mod. Phys.* **89**, 015003 (2017). URL https://link.aps.org/doi/10.1103/RevModPhys.89.015003.

[10] Migdal, A. Interaction between electrons and lattice vibrations in a normal metal. *Sov. Phys. JETP* **7**, 996–1001 (1958).

[11] Eliashberg, G. Interactions between electrons and lattice vibrations in a superconductor. *Sov. Phys. JETP* **11**, 696–702 (1960).

[12] Roy, B., Sau, J. D. & Das Sarma, S. Migdal's theorem and electron-phonon vertex corrections in dirac materials. *Phys. Rev. B* **89**, 165119 (2014). URL https://link.aps.org/doi/10.1103/PhysRevB.89.165119.

[13] Alexandrov, A. S. Breakdown of the migdal-eliashberg theory in the strong-coupling adiabatic regime. *Europhysics Letters (EPL)* **56**, 92–98 (2001). URL https://doi.org/10.1209/epl/i2001-00492-x.

[14] Bauer, J., Han, J. E. & Gunnarsson, O. Quantitative reliability study of the migdal-eliashberg theory for strong electron-phonon coupling in superconductors. *Phys. Rev. B* **84**, 184531 (2011). URL https://link.aps.org/doi/10.1103/PhysRevB.84.184531.

[15] Lee, J. Y., Ahn, J., Zhou, H. & Vishwanath, A. Topological correspondence between hermitian and non-hermitian systems: Anomalous dynamics. *Phys. Rev. Lett.* **123**, 206404 (2019). URL https://link.aps.org/doi/10.1103/PhysRevLett.123.206404.
